# Depth wide distribution and metabolic potential of chemolithoautotrophic microorganisms reactivated from deep continental granitic crust underneath the Deccan Traps at Koyna, India

**DOI:** 10.3389/fmicb.2022.1018940

**Published:** 2022-11-24

**Authors:** Sunanda Mandal, Himadri Bose, Kheerthana Ramesh, Rajendra Prasad Sahu, Anumeha Saha, Pinaki Sar, Sufia Khannam Kazy

**Affiliations:** ^1^Environmental Microbiology and Biotechnology Laboratory, Department of Biotechnology, National Institute of Technology Durgapur, Durgapur, WB, India; ^2^Environmental Microbiology and Genomics Laboratory, Department of Biotechnology, Indian Institute of Technology Kharagpur, Kharagpur, WB, India

**Keywords:** Koyna seismogenic zone, terrestrial subsurface granitic rock, deep biosphere, inorganic carbon metabolism, chemolithotrophic microorganisms

## Abstract

Characterization of inorganic carbon (C) utilizing microorganisms from deep crystalline rocks is of major scientific interest owing to their crucial role in global carbon and other elemental cycles. In this study we investigate the microbial populations from the deep [up to 2,908 meters below surface (mbs)] granitic rocks within the Koyna seismogenic zone, reactivated (enriched) under anaerobic, high temperature (50°C), chemolithoautotrophic conditions. Subsurface rock samples from six different depths (1,679–2,908 mbs) are incubated (180 days) with CO_2_ (+H_2_) or HCO_3_^−^ as the sole C source. Estimation of total protein, ATP, utilization of NO_3_^-^ and SO_4_^2−^ and 16S rRNA gene qPCR suggests considerable microbial growth within the chemolithotrophic conditions. We note a better response of rock hosted community towards CO_2_ (+H_2_) over HCO_3_^−^. 16S rRNA gene amplicon sequencing shows a depth-wide distribution of diverse chemolithotrophic (and a few fermentative) Bacteria and Archaea. *Comamonas, Burkholderia-Caballeronia-Paraburkholderia, Ralstonia*, *Klebsiella*, unclassified Burkholderiaceae and Enterobacteriaceae are reactivated as dominant organisms from the enrichments of the deeper rocks (2335–2,908 mbs) with both CO_2_ and HCO_3_^−^. For the rock samples from shallower depths, organisms of varied taxa are enriched under CO_2_ (+H_2_) and HCO_3_^−^. *Pseudomonas*, *Rhodanobacter*, *Methyloversatilis*, and Thaumarchaeota are major CO_2_ (+H_2_) utilizers, while *Nocardioides*, *Sphingomonas*, *Aeromonas*, respond towards HCO_3_^−^. H_2_ oxidizing *Cupriavidus*, *Hydrogenophilus*, *Hydrogenophaga*, CO_2_ fixing Cyanobacteria *Rhodobacter*, *Clostridium*, *Desulfovibrio* and methanogenic archaea are also enriched. Enriched chemolithoautotrophic members show good correlation with CO_2_, CH_4_ and H_2_ concentrations of the native rock environments, while the organisms from upper horizons correlate more to NO_3_^−^, SO_4_^2−^_,_ Fe and TIC levels of the rocks. Co-occurrence networks suggest close interaction between chemolithoautotrophic and chemoorganotrophic/fermentative organisms. Carbon fixing 3-HP and DC/HB cycles, hydrogen, sulfur oxidation, CH_4_ and acetate metabolisms are predicted in the enriched communities. Our study elucidates the presence of live, C and H_2_ utilizing Bacteria and Archaea in deep subsurface granitic rocks, which are enriched successfully. Significant impact of depth and geochemical controls on relative distribution of various chemolithotrophic species enriched and their C and H_2_ metabolism are highlighted. These endolithic microorganisms show great potential for answering the fundamental questions of deep life and their exploitation in CO_2_ capture and conversion to useful products.

## Introduction

Recent explorations of the deep biosphere in the crystalline basement of continents and oceanic crust have produced a growing awareness on life extending deep interior of the igneous provinces (Nyyssönen et al., 2014; Lau et al., 2016; Dutta et al., 2018, 2019; Magnabosco et al., 2018; Bell et al., 2020; Purkamo et al., 2020; Suzuki et al., 2020; Sahu et al., 2022). The subsurface beneath the Earth’s continental crust is constrained with multiple physical and chemical extremities including high temperature and pressure, lack of sufficient pore space for fluid and organismic mobility, water and readily metabolizable nutrients (Kieft, 2016). Yet, evidence supports life’s presence at depths down to ~1 km beneath seafloor, and ~5 km in continental settings harbouring ~12–20% of the total biomass of microorganisms on Earth, mostly in the continental subsurface (Magnabosco et al., 2018; Purkamo et al., 2020). Previous evidence indicates that terrestrial subsurface holds diverse and novel microbes with potential for answering questions cardinal to the origin, adaptation and evolution of life, their relevance in elemental cycling, and in production of valuable resources (Escudero et al., 2018). However, despite these observations, the deep biosphere has been largely overlooked and the understanding of the metabolic functions, especially mechanisms of carbon and energy metabolism of the deep continental subsurface microbiome, has remained less explored.

The primary energy source in the deep biosphere is proposed to be geochemical (Gold, 1992; Amend and Teske, 2005; Colwell and D’Hondt, 2013). Terrestrial Deep subsurface, having limited photosynthetically derived energy substrates, are fueled primarily by microbial chemotrophy, where versatile chemolithotrophs, capable of inorganic C assimilation, oxidation of H_2_ and S compounds coupled with the reduction of diverse available electron acceptors are often found to be predominant. The existence of a specific hydrogen-driven microbial ecosystem (subsurface lithoautotrophic microbial ecosystems; SLiMEs), energetically powered by the geologically derived sources, is also proposed to drive the oligotrophic subsurface community (Nealson et al., 2005; Osburn et al., 2014; Bell et al., 2020). Rocks themselves can act as the source for different mineral nutrients and ions produced due to high temperature, water-rock interactions and other geochemical reactions. Geogenic supply of CO_2_ and CH_4_ (as C sources), H_2_, CH_4_, NH_4_^+^, Fe^2+^ and S^2−^ (as electron donors) and NO_3_^−^, Fe^3+^, Mn^4+^, SO_4_^2−^ and CO_2_ (as electron acceptors) have potential roles in providing nutrient and energy supplies to the residing community (Colwell and D’Hondt, 2013; Konn et al., 2015; Kazy et al., 2021). Even though the dominance of lithoautotrophic metabolism has been reported from several terrestrial subsurface environments including Fennoscandian shield (Nyyssönen et al., 2014) and Witwatersrand Basin (Lau et al., 2016), the role of heterotrophic organisms, capable of utilizing the metabolic products of autotrophic reactions and driving the overall community function has also been highlighted (Purkamo et al., 2015; Bomberg et al., 2016; Momper et al., 2017; Sahu et al., 2022). Several of these studies have highlighted an intricate and interconnective association of autotrophic and heterotrophic processes in driving the overall communities.

Samples from different subsurface settings have been previously used to investigate the abundance, nature, and substrate specific response of deep dwelling organisms *via* several cultivation-based methods and enrichment studies (Kotelnikova and Pedersen, 1998; Hallbeck and Pedersen, 2008, 2012; Fichtel et al., 2015; Rajala et al., 2015; Russell et al., 2016; Purkamo et al., 2017, 2020; Rajala and Bomberg, 2017; Leandro et al., 2018; Imachi et al., 2019; Sanz et al., 2021; Nuppunen-Puputti et al., 2022). The outcome of these studies, resulted in the enrichment of microorganisms mostly belonging to bacterial phyla Proteobacteria, Firmicutes, Actinobacteria, Chloroflexi and Bacteroidetes (Rastogi et al., 2009, 2013; Fichtel et al., 2012; Fortunato and Huber, 2016; Rajala and Bomberg, 2017; Leandro et al., 2018) and archaeal members including Euryarchaeota, Thaumarchaeota and Crenarchaeota (Zeng et al., 2009; Nuppunen-Puputti et al., 2018; Imachi et al., 2020; Alain et al., 2021; Courtine et al., 2021; Li et al., 2021). “In the last few years, metagenomic investigations of terrestrial subsurface microbiome” (crustal/vent, faults or fractures fluids, aquifer samples have shown considerable species diversity with potential for assimilation of C and inorganic resources for energy metabolism (Nyyssönen et al., 2014; Purkamo et al., 2015; Hubalek et al., 2016; Lau et al., 2016; Magnabosco et al., 2016; Wu et al., 2016; Momper et al., 2017; Bell et al., 2020; Purkamo et al., 2020). In contrast to subsurface fluid-based studies, reactivation of inhabitant microbiota of Archean basement rocks has remained relatively less explored. Recently, deep subsurface studies on rock cores from Iberian Pyrite and a few other crystalline bedrocks and basalt samples from Deccan Traps have been carried out (Dutta et al., 2018, 2019; Leandro et al., 2018; Purkamo et al., 2020; Sanz et al., 2021; Sahu et al., 2022). However, reactivation and enrichment of deep endolithic microorganisms of the granitic bedrock are inadequately studied and their metabolic capabilities remain less known.

Scientific continental drilling of deep boreholes around the Koyna seismogenic zone (KSZ) in the southwestern part of the Deccan Volcanic Province (DVP) enabled a rare access to the basement beneath the Deccan Traps, the nature of which has been enigmatic for longtime (Roy et al., 2013; Roy, 2017). The Deccan Traps (~65 Ma) represents a massive continental flood basalt province comprising of erupted lavas and associated rocks that rests on 2.5 Ga crystalline basement (Schoene et al., 2015; Bhaskar Rao et al., 2017; Dutta et al., 2018). The Koyna–Warna region has gained substantial research interest owing to the intermittent reservoir-induced seismic activity that started after the impoundment of the Shivajisagar (Koyna) artificial water reservoir in 1962 (Gupta, 2017). A scientific borehole (3 km deep, named as Koyna pilot borehole, KFD1), was drilled in 2017 for setting up a deep borehole observatory in the region (Goswami et al., 2020). Rock cores obtained from various depth intervals (1,679–2,908 mbs) through this borehole were used to reactivate and enrich microorganisms under chemolithoautotrophic conditions. The specific goals for this study were to (i) understand the response of endolithic microbes (from different depths) towards the growth substrates CO_2_ (along with H_2_ as electron donor) or HCO_3_^−^ as the sole C source (ii) identify the nature of reactivated microbial community members through amplicon based 16S rRNA gene sequencing and to assess their depth-wide distribution pattern, and (iii) elucidate the metabolic potential of the enriched microbial communities, through predicted metabolic profiling, with specific emphasis on C and energy metabolism processes.

## Materials and methods

### Sample collection and rock processing

Subsurface rock core samples were obtained from different depths (1,679–2,908 mbs) of the Koyna pilot borehole (17°17″57.27” N, 73°44″19.07″ E) of the Deccan Traps, India. Samples were collected following standard techniques and stored anaerobically in sterile containers at 0–4°C and aseptically transported to the laboratory. The detailed processes of handling and storage procedures of the samples were mentioned in the previous study from our group (Sahu et al., 2022). For microbiological investigations, rocks were powdered in N_2_-filled condition, using electrical coring machine, and the interior rock pieces/powder were obtained (Sahu et al., 2022). Details of the six rock samples (designated as C1, C2, C3, C4, C6 and C7), have been furnished in Supplementary Table 1, and were used for further enrichment studies.

### Growth medium, culture conditions and reactivation of indigenous microorganisms

Reactivation of indigenous microorganisms from the six rock core samples was performed by setting up microcosm-based enrichment setups. The rock powder was checked for the presence of contamination by estimating the sodium fluorescein concentration (used as a marker of potential contamination of drilling fluid) as per the protocol described earlier (Dutta et al., 2018). Rock powders devoid of any sodium fluorescein were used for further analysis. 0.05 g of rock powder samples were incubated anaerobically (in crimp sealed serum vials) at high temperature (50°C) using a basal medium [formulated based on the overall geochemistry of our rock samples as reported in (Sahu et al., 2022) and named as the Koyna medium] and other supplements. A Don Whitley anaerobic workstation (Model A-35) operating with N_2_/H_2_ + CO_2_ + N_2_ (10:10:80) gas environment was used to setup the enrichments. The detailed composition of the medium and incubation conditions, etc. have been provided in Supplementary Table 2. All medium components were prepared separately, sterilized either by autoclaving or filtration, (as appropriate) and used. The pH of the medium was set at pH 9.0. For the present study, each of the six samples (C1, C2, C3, C4, C6 and C7) were incubated with CO_2_ (along with H_2_ as electron donor) or 2 mM HCO_3_^−^ (along with NH_4_ as electron donor) as sole C source. CO_2_ was added from a cylinder containing high purity gas mixture of CO_2_ and H_2_ in a premixed concentration ratio of 80:20. Ten ml of the gas mixture was added using the liquid displacement technique. A total of 12 experimental setups [marked as C1–C7 HC (for H_2_ + CO_2_ enriched sets) and C1–C7 BC (for HCO_3_^−^ enriched sets)] and two controls (setups devoid of rock samples) were incubated for 180 days at 50°C and considered for further study post-incubation.

### Measurement of growth parameters: Protein estimation

Total protein concentration of the enrichment cultures was measured to estimate the microbial biomass content. Protein concentration was measured through a fluorometric method (Qubit, Thermo-Fisher Scientific), using the Qubit Protein Assay Kit. In brief, enrichment samples were sonicated with 30% amplitude and pulse of 15 s on and 15 s off, for 10 min in order to obtain the total protein from the enrichment cultures. After sonication, the solution was centrifuged at 10,000 rpm for 5 min and the supernatant was collected and used as test samples for the estimation. The working solution as well as assay tubes were prepared and the estimation was done in triplicate using the manufacturers protocol (https://research.fredhutch.org/content/dam/stripe/hahn/methods/mol_biol/Qubit_Protein_Assay_QR.pdf). The fluorometer was calibrated against the standards and the test samples were measured in μg ml^−1^.

### Measurement of growth parameters: ATP estimation

A Luminometer (Glomax 20/20 System, Promega with BacTiter-Glo™ Microbial Cell Viability Assay kit) was used for estimating the total ATP of the living cells present in the enrichment samples (Adhikari and Kallmeyer, 2010). The details of the protocol were same as provided by the manufacturer, *i.e* the BacTiter-Glo™ Microbial Cell Viability Assay Technical Bulletin #TB337. Light emission was measured (as relative light units per second, RLU s^−1^) for three, five seconds intervals with a five seconds delay before each interval, which was then quantified as mM with reference to standard ATP curve. Sterile tips were used in transferring all solutions and samples to exclude ATP contamination of pipettes and solutions. All procedures were performed in triplicates in the dark and all plastic material, solutions, and pipettes were stored in the dark as light could hamper the results causing delayed fluorescence.

### Measurement of growth parameters: Utilization of terminal electron acceptors

The utilization/reduction of electron acceptors might act as a significant sign of growth of microorganisms. In this study, the initial and final concentrations of terminal electron acceptors like SO_4_^2−^ and NO_3_^−^, were measured to obtain information about the change in their concentration. Required amounts of enrichment cultures were obtained, centrifuged at 10,000 rpm for 30 min and the supernatant was used for further analysis. SO_4_^2−^ estimation was performed through the BaCl_2_ based turbidimetric spectroscopy method (Chesnin and Yien, 1951). For measurement of NO_3_^−^ concentration, the samples were treated with salicylic acid-H_2_SO_4_ reagent, followed by NaOH, and estimated *via* colorimetric procedures (Cataldo et al., 1975). All analyses were performed in triplicates.

### Measurement of metabolite concentration

Formation of different metabolites as intermediates or end-products of various pathways could provide an indication of successful utilization of the provided enrichment substrates. Metabolites such as lactate, acetate, and formate in the enrichment setups were measured through Ion Chromatography (Eco IC, Metrohm) with PRP-X300 (Hamilton) column following the manufacturer’s protocol. The standard solutions were prepared using highly purified grade sodium salts. 15 ml culture was aseptically and anaerobically withdrawn from each of the serum vials, centrifuged (10,000 rpm for 30 min) and the supernatant was used for IC analysis. The samples were acidified using 0.1 N HCl, filtered using 0.22 μm filter (Merck Milipore, Millex GP) and transferred to the autosampler for further processing.

### DNA extraction and 16S rRNA gene amplification and sequencing

The entire culture volume of the serum vials (~100 ml) from each enrichment was centrifuged at 10,000 rpm for 30 min and the pellet (containing the rock powder and microorganisms) was used for extraction of total DNA in multiple replicates (*n* = 4) using QIAGEN power soil DNA isolation kit with certain modifications (30 min incubation after adding C2 and C3 solutions each). The total volume of eluted DNA from each sample was pooled, concentrated and quantified through a fluorometric method (Qubit, Thermo-Fisher Scientific). The V4 region of 16S rRNA gene was amplified from the isolated total DNA with V4-specific barcoded primers 515F (5′-GTGCCAGCMGCCGCGGTAA-3′) and 806R (5′-GGACTACVSGGGTATCTAAT-3′, Bates et al., 2011). The V4 amplicon libraries were purified, processed and sequenced through an in-house Ion S5 (Thermo-Fisher Scientific). Details of the sequencing procedures are described elsewhere (Dutta et al., 2018). Total DNA was extracted from sequencing reagent and sequenced using the same procedure, as control. The sequence reads were submitted to the Short Read Archive (NCBI) under BioProject ID PRJNA642293 (for H_2_ + CO_2_ set) and PRJNA643233 (for HCO_3_^−^ set).

### Quantitative polymerase chain reaction

Bacterial abundance in the enrichment cultures was quantified by estimating the copy number of bacterial specific 16S rRNA genes (V3 region). All the amplification reactions were set up in triplicate. Quantitative PCR was performed in QuantStudio 5 using Power SYBR green PCR master mix (Invitrogen), with primer concentration of five pM and the following amplification conditions: 95°C for 10 min, 40 cycles of 95°C for 15 s, 55°C for 30 s and 72°C for 30 s. Melting curve analysis was run after each assay to check PCR specificity. The primers used were P1 (5’-CCTACGGGAGGCAGCAG-3′) and P2 (5’-ATTACCGCGGCTGCTGG-3′). Other details are same as described earlier (Dutta et al., 2018). Samples were run in at least three dilutions to check for PCR inhibitions. Bacterial 16S rRNA gene copy numbers were determined in each sample by comparing the amplification result to a standard dilution series ranging from 10^2^ to 10^10^ of plasmid DNA containing the V3 region of the 16S rRNA gene of *Achromobacter* sp. MTCC 12117. We could not perform the quantification (as we did not obtain any amplification) of archaeal populations, because of lower DNA concentrations.

### Quality filtering, operational taxonomic unit picking and annotation

Raw sequence reads obtained after sequencing were quality filtered (removal of primers, homopolymers run of less than six bp, read length beyond the range of 250–300 bp, mean quality score below minimum of 25, with minimum six primer mismatches) and analysed in QIIME 1.9.1 pipeline (Caporaso et al., 2010). Denovo-based clustering (97% sequence similarity) of filtered reads to OTUs was performed using UCLUST under QIIME workflow. Taxonomic assignment of representative reads from each OTU was performed using SILVA 132 database.^1^ The taxonomic assignments could not be performed in newer versions of SILVA database due to unavailability of required computational facilities. OTUs detected in reagent control, singleton OTUs (the cumulative read for which is one amongst all sets, Reitmeier et al., 2021) and OTUs affiliated to potential contaminants of deep subsurface, i.e., members of taxa Staphylococcaceae, Streptococcaceae, Propionibacteriaceae, Lactobacillaceae, *E. coli-Shigella, Corynebacterium* (Hubalek et al., 2016; Sheik et al., 2018), were removed from the OTU table before further analysis. Samples were normalized to 14,800 reads by random sampling for alpha diversity analysis.

### Statistical analyses

Principal Component Analysis (PCA) and Canonical Correspondence Analysis (CCA) were performed using PAST software version 4.06 (Hammer et al., 2001) on the basis of (i) growth parameters and (ii) concentration of selected geochemical parameters along with abundant taxa (top 50% relative abundance) in both enrichment sets, respectively. Non-metric multidimensional scaling analysis (NMDS, Dixon, 2003; Doi and Okamura, 2011) was performed for comparing the similarity/dissimilarity of the microbial communities at taxonomic level by using metaMDS function of ‘vegan’ package in R software 4.2.1 (Kuhnert et al., 2005; Van der Loo, 2012) which helped to depict the effect of different substrates and depth on the microbial communities. Similarity percentage (SIMPER) analysis was done by using Simper function in ecological vegan package of R software 4.2.1 (R Development Core Team, 2017) to identify the microbial taxa responsible for the dissimilarity between different depths in both the substrate types (HC and BC). OTU overlap among the enrichments from different substrates and among different rocks in the same enrichment were elucidated using InteractiVenn (Heberle et al., 2015). Phyla belonging to individual enrichments were selected for correlation analysis and the correlation heatmap was constructed on the basis of Spearman correlation using METAGENassist (Arndt et al., 2012).

### Phylogenetic analysis

16S rRNA sequence reads of various OTUs were used to construct a phylogenetic tree using a distance matrix-based Neighbor-joining method in MEGA 11 software with 1,000 bootstrap re-iterations (Tamura et al., 2021). Initially, top 15 abundant OTUs from each enrichment set and the OTUs affiliated to Cyanobacteria from the enrichments were analysed by homology search for the closest sequence relatives (matches) using the National Centre for Biotechnology Information (NCBI) BLASTn nucleotide database. Multiple sequence alignments were performed with CLUSTALW package of MEGA 11. The sequences were represented with their accession number followed by the organism’s identity and its location. Finally, phylogenetic tree reconstruction and validation were performed using Neighbor-Joining (NJ) algorithm (Saitou and Nei, 1987) with 1,000 bootstrap re-iterations using Jukes-Cantor distance model (Jukes and Cantor, 1969).

### Network analysis

Co-occurrence network analysis was performed at two different levels, considering (i) OTUs enriched in at least four samples out of total six at the lowest taxa level and (ii) association of taxa strongly correlated with depth of the rock samples and with relevant geochemical parameters of the rocks. Correlation among different taxa was calculated using otu.association command in mothur (Schloss et al., 2009). Correlation among different taxa and geochemical parameters was calculated using the PAST software version 4.06 (Hammer et al., 2001). The Spearman correlation values r ≥ ±0.9 and r ≥ ±0.3 were used for construction of co-occurrence network amongst taxa and amongst taxa and parameters, respectively. Visualization of the interaction network was done using Cytoscape 3.7.1 (Shannon et al., 2003).

### Metabolic characterization of enrichment sample through PICRUSt v2 (Phylogenetic Investigation of Communities by Reconstruction of Unobserved States) analysis

Microbial metabolic pathways were estimated based on the 16S rRNA gene data from the closed OTU picking method using the PICRUSt v2 software (Douglas et al., 2020). PICRUSt v2 compares 16S rRNA marker gene data with the nearest match of the known genome sequence to allocate the functional attributes. A weighted nearest sequenced taxon index (NSTI) was calculated to evaluate the accuracy of prediction. The predicted KO numbers were plotted on KEGG pathway maps^2^ separately. Abundances of genes related to general metabolic pathways, different C fixation pathways, lithotrophy related metabolisms, secondary metabolite production and xenobiotic degradation were selected for detailed analysis.

## Results

### Microbial response in enrichment microcosms

**Figure 1:**
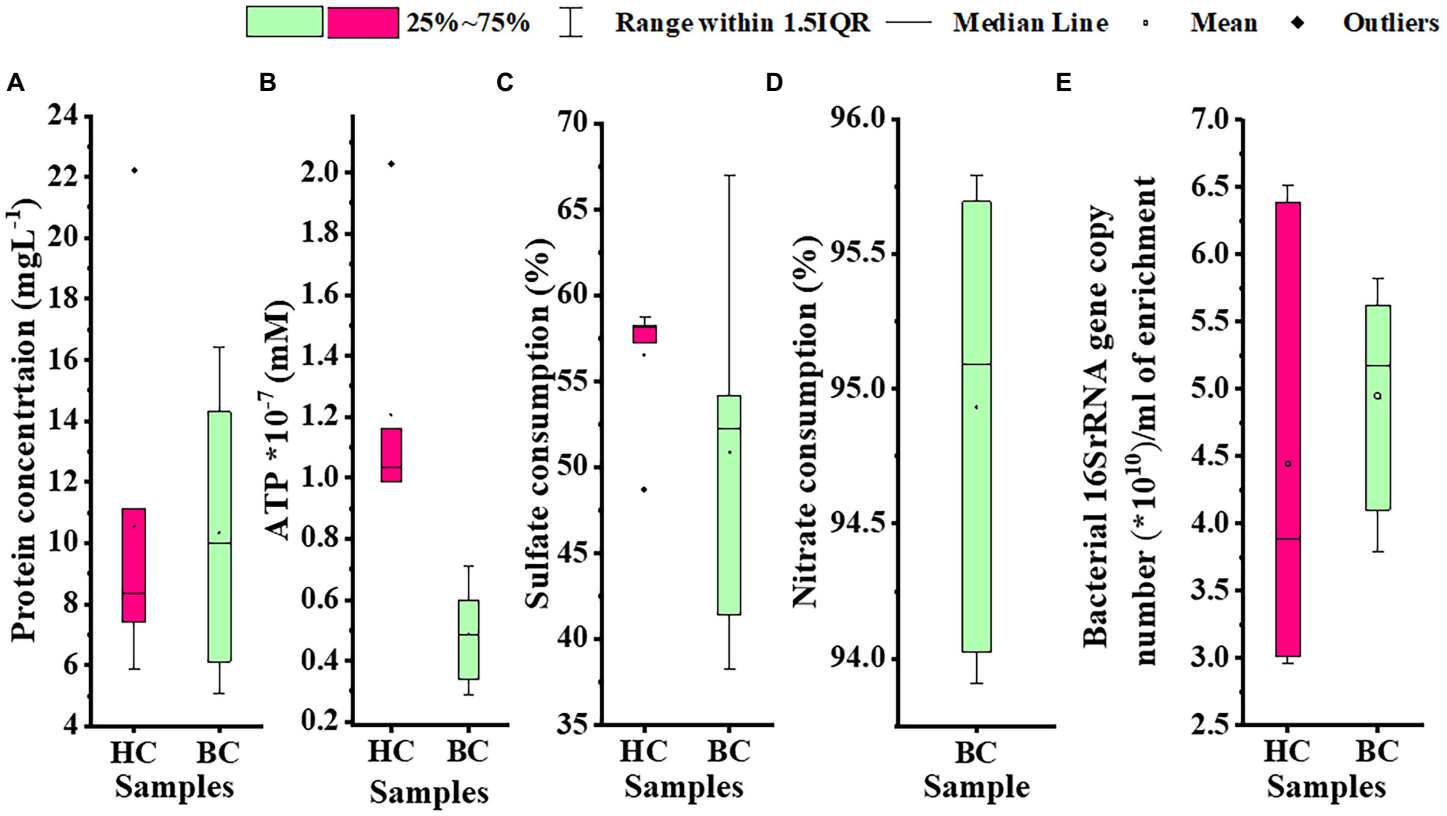
Assessment of **(A)** cell biomass in terms of total protein, **(B)** ATP, **(C)** utilization of sulfate; provided as TEA, **(D)** utilization of nitrate; as TEA, **(E)** Bacterial 16S rRNA gene copy number per mL of enrichment in HC and BC sets. The black center line denotes the median value (50th percentile), the empty square denotes the mean value and the pink (HC) and green (BC) boxes contain the 25–75th percentiles of the data sets. The values beyond these upper and lower boundaries are considered as outliers, marked with black filled diamond shapes.

**Figure 2:**
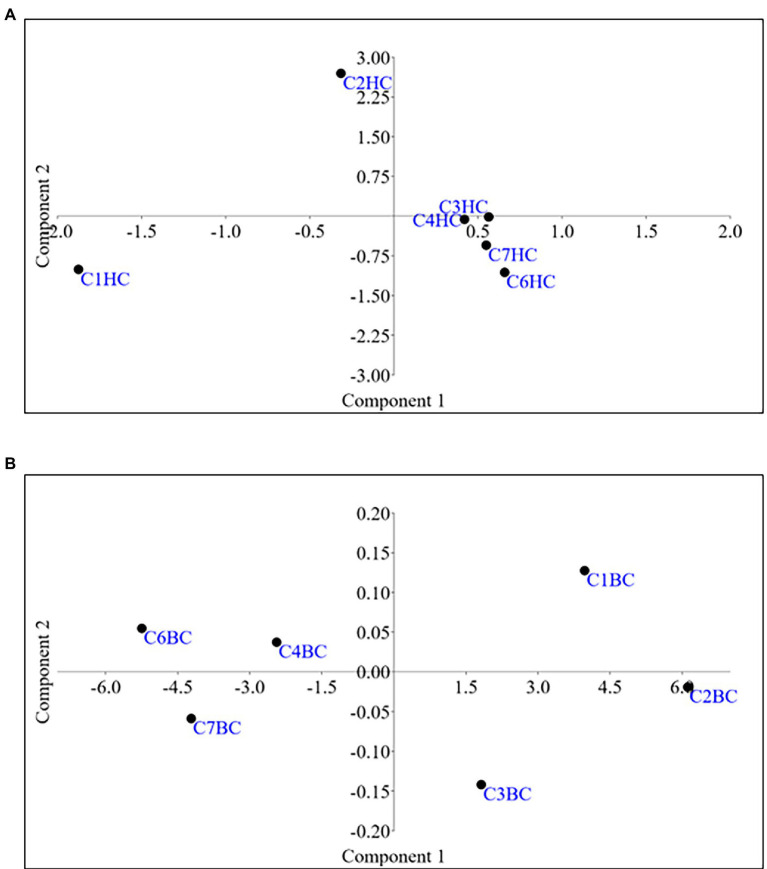
Principal Component Analysis for **(A)**HC/**(B)**BC enrichments based on the growth parameters.

Microbial enrichment and response in anaerobic high temperature enrichment microcosms was assessed by estimating the cell biomass in terms of total protein, ATP, as a metabolic marker, utilization of SO_4_^2−^ and NO_3_^−^ (provided as terminal electron acceptor) and bacterial 16S rRNA gene copy numbers (Figures 1A–E). Substantial amounts of protein and ATP were obtained in both setups indicating a positive response of the rock communities to the substrate provided. Consumption of ~50% of the initial SO_4_^2−^ concentration or ~ 94% of the initial NO_3_^−^ concentration could be observed. The total amount of bacterial gene copy number in each enrichment culture as estimated by 16S rRNA gene targeted qPCR analysis indicated 2.9 × 10^10^ ml^−1^ to 6.5 × 10^10^ ml^−1^ copies in HC enrichment while in BC enrichments it ranged from 3.7 × 10^10^ ml^−1^ to 5.8 × 10^10^ ml^−1^. However, archaeal 16S rRNA genes could not be amplified during qPCR analysis. A PCA biplot on the basis of observed growth parameters reflected the distribution of the rock core enrichment samples in two halves of the plot (Figures 2A,B).

### Formation of metabolites

Identification of different metabolites (small organic acids), *via* ion chromatography was done. Approximately 77 mgl^−1^ and 1.0 mgl^−1^ formate as well as 191.1 mgl^−1^ and 6.6 mgl^−1^ lactate in HC and BC microcosms, respectively were estimated. However, ~9.5 mgl^−1^ acetate was detected only in the BC sets (Supplementary Table 3).

### Sequence details and alpha diversity indices

Details of the sequence information have been summarized in Table 1. A total of 389,051 (HC) or 86,100 (BC) 16S rRNA reads was obtained. In HC setup, a maximum of 141,124 and a minimum of 18,136 quality filtered reads were obtained from C1HC and C4HC sets, respectively. Whereas in case of BC enrichments, a maximum of 23,686 and a minimum of 4,400 quality filtered reads were obtained from C7BC and C3BC sets, respectively. On an average more than 99% of the quality filtered reads could be classified as bacteria (368,807 in HC and 85,124 in BC sets) and archaea (16,989 in HC and 286 in BC sets). Following the removal of control and potential erroneous reads, a total of 18,258 OTUs (HC) and 10,482 OTUs (BC) were obtained (Supplementary Figure 1). The Venn diagram illustrated in Supplementary Figures 2A,B depicted the sharing of OTUs amongst the six samples in each enrichment. It was observed that the percentage of unique OTUs and their cumulative abundance decreased as the depth of the rock (used as sample for enrichment) increased. Only 34 and 9 OTUs were common amongst all six samples in HC and BC enrichments, respectively.

**Table 1:** Sequencing information of samples in H_2_ + CO_2_ (designated as C1–C7 HC) and HCO_3_^−^ (are designated as C1–C7 BC) enrichment samples.

Samples	Number of quality filtered reads	Number of reads classified	Number of OTUs (w/o singletons)	No. of phyla bacteria/Archea	No. of families bacteria/Archea	No. of genera bacteria/Archea
C1HC	141,124	138,922 (98.4%)	6,249	26/4	193/6	339/4
C2HC	132,911	132,616 (99.7%)	5,387	22/2	151/2	258/1
C3HC	54,468	53,945 (99.03%)	4,480	31/4	210/10	370/6
C4HC	18,136	18,104 (99.8%)	4,051	13/1	97/1	156/1
C6HC	21,415	21,249 (99.2%)	4,727	27/4	149/8	244/6
C7HC	20,997	20,960 (99.82%)	4,304	12/1	53/0	86/0
C1BC	6,615	6,567 (99.27)	1,014	17/2	105/1	160/1
C2BC	14,159	13,982 (98.7%)	1,379	20/2	116/3	198/1
C3BC	4,400	4,363 (99.1%)	591	17/0	127/0	160/0
C4BC	18,085	17,971 (99.3%)	4,452	12/1	79/0	121/0
C6BC	19,155	18,997 (99.1%)	4,429	20/3	127/1	205/2
C7BC	23,686	23,530 (99.3%)	5,112	17/2	127/2	192/2

Details of the alpha diversity indices were summarized in Table 2. Chao1 estimator, which estimated the minimum total number of OTUs, ranged from ~4176 (C2HC) to ~7629 (C6HC) in HC sets and 770 (C3BC) to 7236 (C7BC) in the BC microcosms. The Shannon diversity index, which was calculated to evaluate species abundance and evenness in each sample, showed values between 8.4 (C2HC) to 10.1 (C4HC) in HC enrichments, whereas in case of BC enrichments, the values ranged between 7.8 (C3BC) to 10.3 (C7BC). The Shannon index values were slightly higher in the deeper horizon samples (C4–C7) under both enrichment conditions. Simpson index, an indicator of species dominance in a community, ranged between 0.98–0.99 across all enrichment samples.

**Table 2:** Alpha diversity indices for H_2_ + CO_2_ (designated as C1–C7 HC) and HCO_3_^−^ (are designated as C1–C7 BC) enrichment samples.

Samples	Chao1	Goods coverage	Shannon index (H′)	Simpson index (λ)	ACE
C1HC	4766.65	0.91	8.88	0.99	4807.51
C2HC	4176.03	0.93	8.45	0.99	4553.18
C3HC	4684.003	0.91	9.09	0.99	4752.75
C4HC	6236.15	0.87	10.18	0.99	6709.35
C6HC	7429.3	0.85	9.79	0.98	7774.59
C7HC	5821.09	0.87	9.74	0.98	6299.06
C1BC	2058.12	0.92	8.14	0.98	2150.93
C2BC	2939.65	0.95	8.44	0.99	3212.48
C3BC	770.93	0.95	7.82	0.99	808.06
C4BC	6658.15	0.86	9.89	0.98	7228.79
C6BC	6828.39	0.86	10.10	0.99	7492.57
C7BC	7236.27	0.85	10.33	0.99	7780.13

### Microbial community compositions in enrichment microcosms

The details of sequence affiliations in each taxonomic level were presented in Table 1. Assigned reads from HC enrichments (total number: 385796) were assigned to 576 genera belonging to 41 phyla (37 bacteria and 4 archaea). Compared to HC, number of taxa detected in BC setups were less. Assigned reads from the BC enrichments (85410) were affiliated to 435 genera belonging to 32 phyla (29 bacteria and 3 archaea).

Members of the phylum Proteobacteria constituted the most dominant group (upto ~63%) across the enrichment conditions (Supplementary Figure 3). The other abundant phyla were Actinobacteria (11%,), Firmicutes (8%), Bacteriodetes (8%), Thaumarchaeota (1.3%), Verrucomicrobia (1.13%), Chloroflexi (2%), Acidobacteria (1.2%) and Cyanobacteria (0.7%). Interestingly, it was observed that for both the enrichment conditions relative abundance of Gammaproteobacteria* (excluding Betaproteobacteriales) and Betaproteobacteriales members were higher in the deeper rock (C4–C7) based enrichments (28 and 53%) than in relatively shallower rock (C1–C3) enrichments (13.5 and 10%). However, the distribution of Actinobacteria, Firmicutes, Bacteroidetes, Cyanobacteria were found to be more in the upper horizon samples (C1–C3HC; C1–C3BC). Members of the phylum Chloroflexi were found to be one of the dominant groups with ~13% abundance in C3BC, whereas Acidobacteria members were found to be relatively abundant in sample C3, in both the HC and BC enrichments.

**Figure 3:**
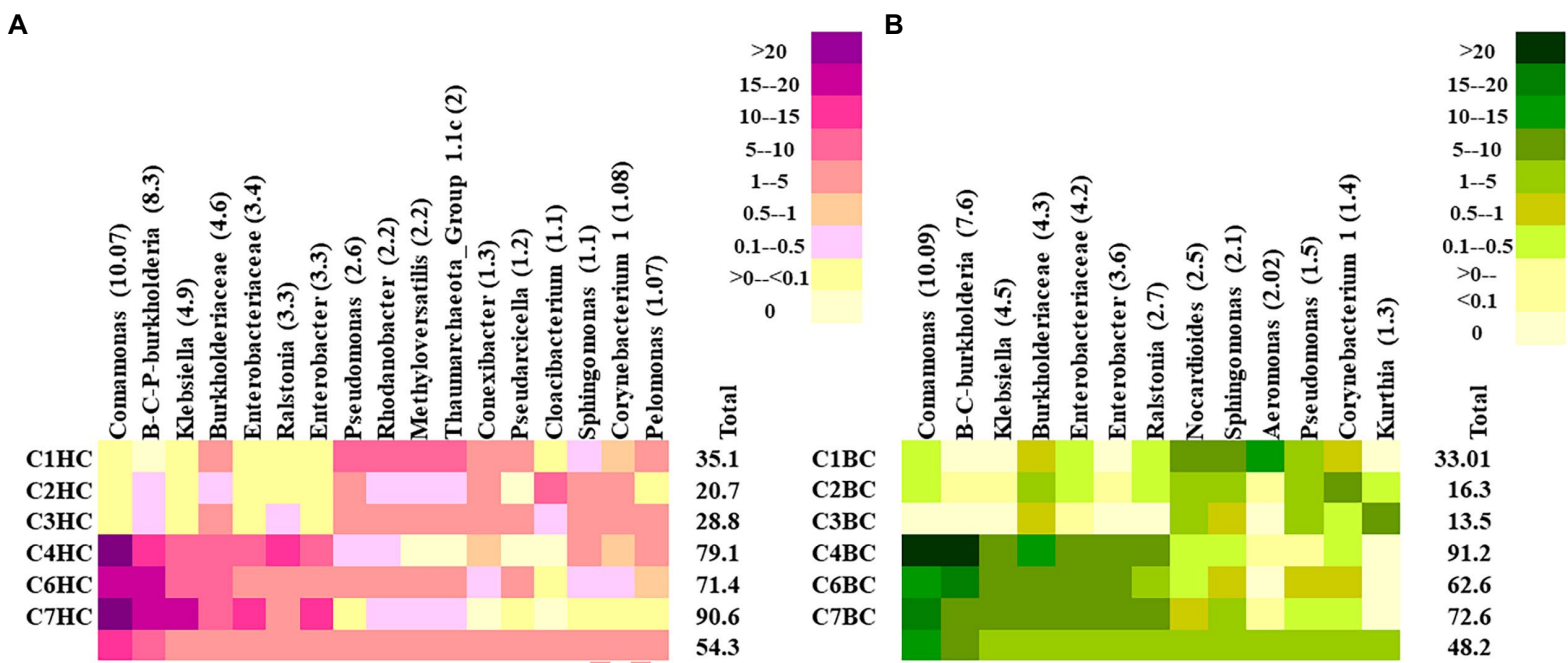
Heatmap displaying the relative abundance of major taxa (abundance ≥1%) detected in **(A)** HC and **(B)** BC enrichments*. Burkholderia-Caballeronia-Paraburkholderia* is designated as B-C-P-burkholderia.

At the lowest taxonomic level, the most abundant microbial groups in HC enrichments were found to constitute >45% of the total community in each setup (Figure 3A). *Comamonas,* was found to be the major taxon in HC enrichments. Other major taxa included the members of *Burkholderia-Caballeronia-Praburkholderia*, *Klebsiella*, *Ralstonia*, *Enterobacter*, unclassified Enterobacteriaceae and Burkholderiaceae. Interestingly, these taxa were more abundant in the rock enrichments from the deeper horizons (C4–C7). On the other hand, mixotrophic *Pseudomonas, Rhodanobacter, Methyloversatilis*, members of Group 1.1c Thaumarchaeota and *Conexibacter* members were more abundant in enrichments of shallower rocks with HC (C1–C3). Noticeably, presence of metabolically versatile Oxyphotobacteria (belonging to Cyanobacteria) was observed in these enrichments with an average relative abundance of 0.7%, with higher abundance in the shallower samples (1.25%). In BC enrichments the top taxa contributed around ~45% in average (Figure 3B). The major groups belonged to similar taxa as in HC enrichment of the deeper horizon rocks. *Comamonas*, *Burkholderia-Caballeronia-Paraburkholderia*, *Klebsiella*, unclassified Enterobacteriaceae, Burkholderiaceae, *Enterobacter* and *Ralstonia* were the most dominant taxa with higher distribution in enrichments from deeper rocks. *Nocardiodes*, *Sphingomonas, Aeromonas* dominated the BC enrichments of shallower rock samples. Other less abundant taxa (relative abundance <0.1%) in HC enrichment microcosms showed the presence of important hydrogen oxidizing and/or CO_2_ utilizing taxa like Chloroflexi, *Curvibacter, Hydrogenophaga, Hydrogenispora, Methanobacterium, Methanolinea, Acidithiobacillus* and *Thiobacillus* as well as desiccation and radiation resistant, endolithic, extremophilic *Chroococcidiopsis*. Syntrophic, chemolithoautotrophic, sulfur oxidizing groups belonging to *Syntrophobacter,* facultatively chemolithoautotrophic *Thermincola, Pelobacter* and autotrophic *Curvibacter*, aromatic compound degrader *Azoarcus* were observed as minor groups in BC enrichment setups. Supplementary Figures 4A,B illustrates the relative abundances of 50 representative less abundant taxa in HC and BC enrichments, respectively.

**Figure 4:**
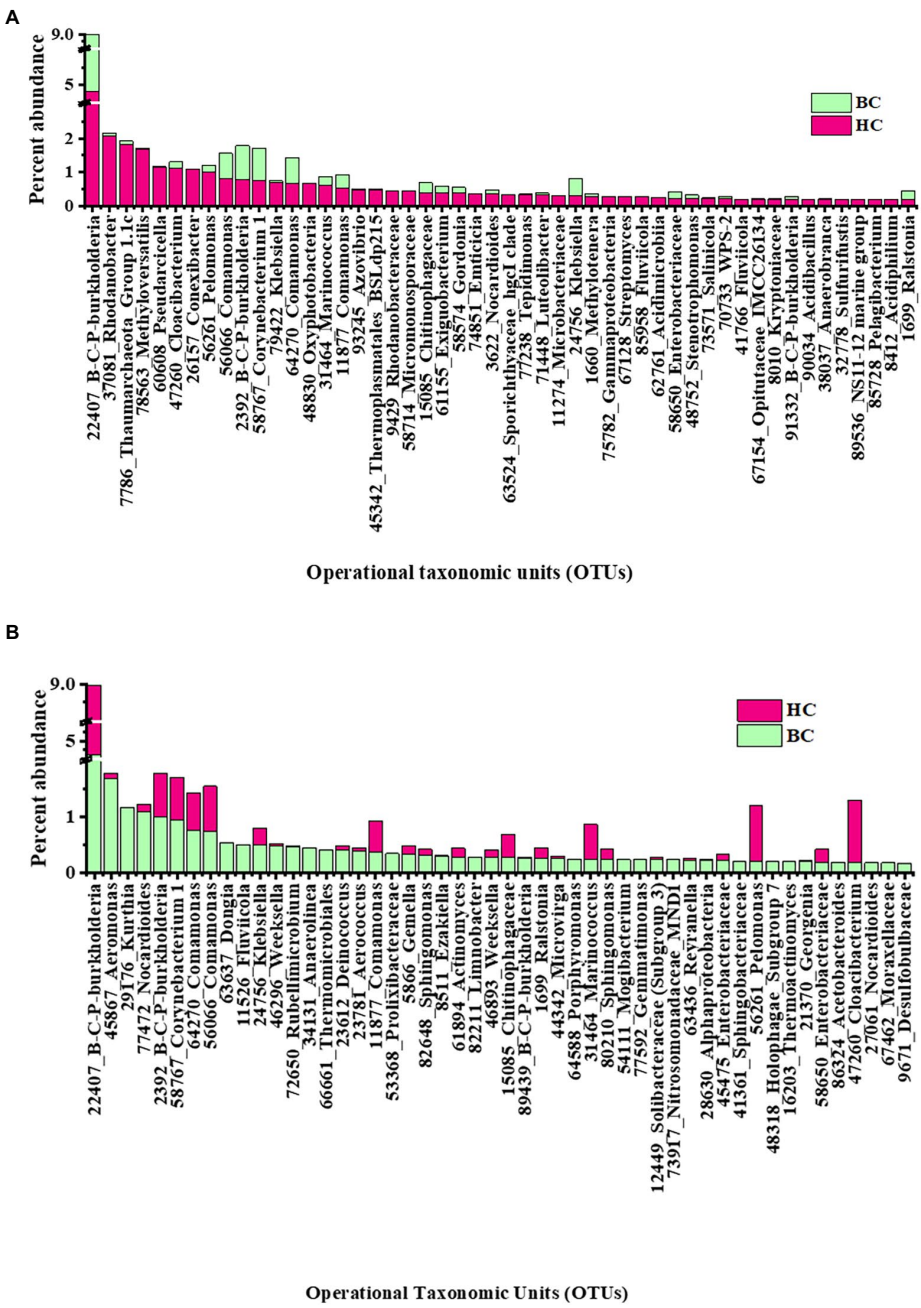
Rank abundance (based on relative percentage abundance) of top 50 OTUs detected in **(A)** HC and **(B)** BC enrichments. *Burkholderia-Caballeronia-Paraburkholderia* is designated as B-C-P-burkholderia.

Rank abundance of observed OTUs was analysed to get an idea of the dominant reactivated community members under different enrichment conditions. In HC enrichments the top 50 OTUs cumulatively contributed to 30% abundance of the total. Rank abundance histograms and taxonomic distribution showed that an OTU, belonging to *Burkholderia-Caballeronia-Paraburkholderia* was the most abundant one with an average relative abundance of 4.8% (Figure 4A). It was followed by the OTUs related to denitrifying *Rhodanobacter* (2.1%), *Thaumarchaeota* (1.8%), *Methyloversatilis* (1.7%), *Pseudarcicella* (1.2%), *Cloacibacterium* (1.1%), taxa capable of hydrogen oxidation like *Conexibacter* (1%) and *Pelomonas* (1%). Interestingly, the presence of nine of these top 10 OTUs mentioned (except *Conexibacter*) were observed to have been enriched with bicarbonate, as enrichment substrate, as well. The other less abundant group contained OTUs belonging to potential H_2_ and CO_2_ utilizing autotrophic organisms like *Ralstonia*, Chloroflexi_KD4-96, Acidobacteraceae (Subgroup 1*), Comamonas, Luteolibacter, Methylobacterium,* Enterobacteraceae and facultative anaerobic Sporichthyaceae.

In BC enrichment microcosms, rank abundance analysis (Figure 4B) of top 50 OTUs, cumulative abundance 24%, revealed that the most abundant OTU to be the same (affiliated to facultative chemolithotrophic *Burkholderia-Caballeronia-Paraburkholderia*) as in the HC enrichment. OTUs related to *Aeromonas* (1.6%), *Kurthia* (1.1%), *Nocardiodes* (1.1%) and *Burkholderia-Caballeronia-Paraburkholderia* (1.01%) followed the dominant one. OTUs related to *Aeromonas, Pseudomonas*, Burkholderiaceae, *Aquabacterium,* Rhodanobacteraceae *Enterobacter, Klebsiella, Nakamurella, Lautropia*, constituted the less abundant OTUs. Supplementary Figures 5A,B illustrates the rank abundances of 50 representative less abundant (relative abundance <0.1%) OTUs in HC and BC enrichments, respectively.

### Statistical analyses

**Figure 5:**
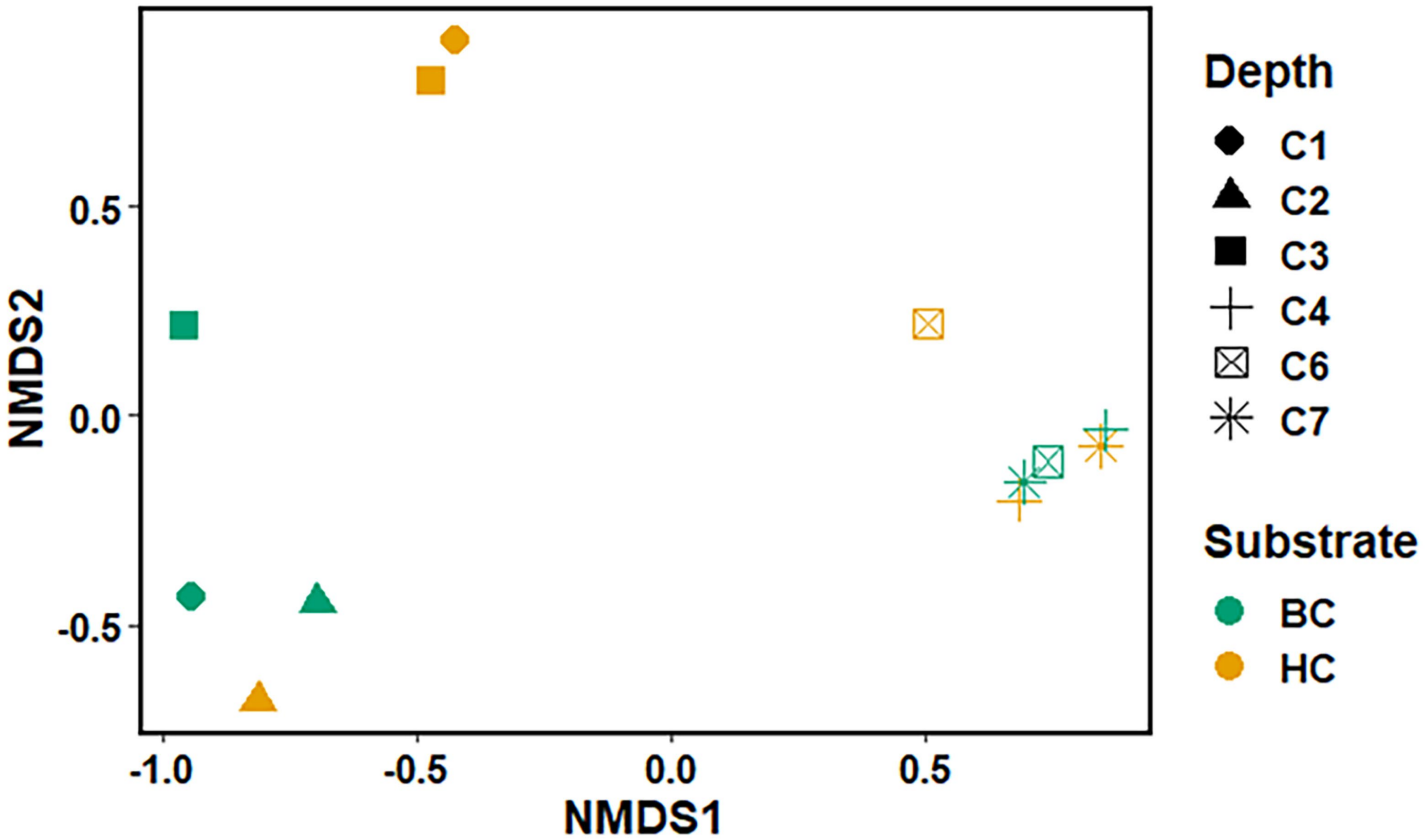
Non-metric Multidimensional scaling of microbial community (at the taxa level) for microbial taxa belonging HC and BC enrichment based on their association with depth.

**Figure 6:**
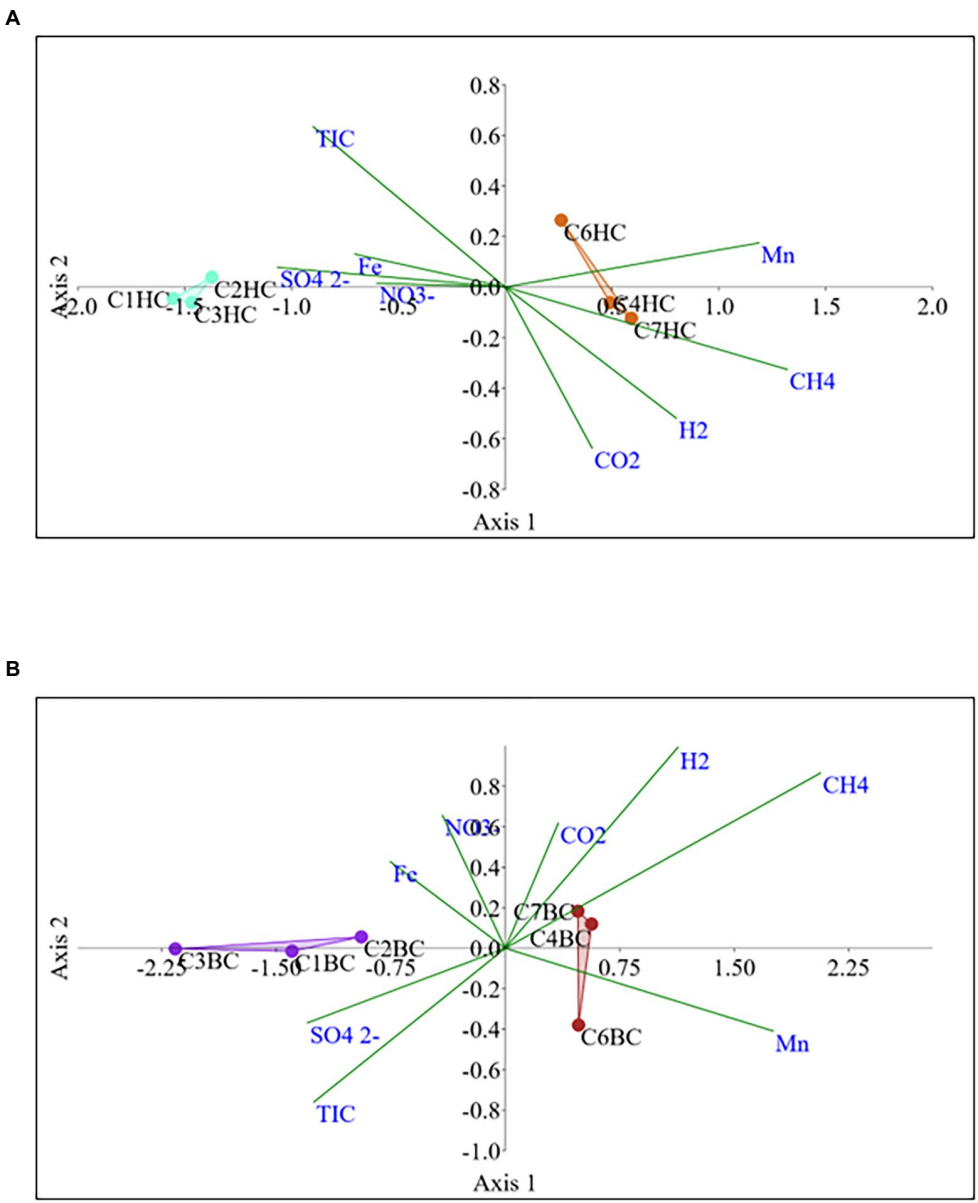
Canonical correspondence analysis for microbial taxa belonging **(A)** HC and **(B)** BC enrichment based on their association with depth and relevant geochemical parameters.

The Non-metric Multidimensional scaling (NMDS) ordination was performed to understand the collective role of enrichment substrates and rock sample’s depths on the distribution of microbial taxa. The output reflected that deeper (C4–C7) rock hosted enriched communities occupied a distinct ordination space separated from shallower (C1–C3) rock enriched communities for each substrate (Figure 5). It was observed that microbial communities were clustered based on depth rather than the enrichment substrates (HC or BC). Stress was found to be ≤0.06 which indicated good fit of ordination (Dexter et al., 2018; Mullin et al., 2020). The partitioning was observed clearly in between the two halves of the plot. A canonical correspondence analysis (CCA) based on selected geochemical parameters along with enriched microbial taxa was drawn (Figures 6A,B), which revealed strong significant correlation of enriched microbial taxa with depth and parameters. Separate guilds were formed which were found to be guided by depth and geochemical parameters. Investigation of the individual enrichment revealed positive correlation of Mn with CCA axes 1 and 2 in HC enrichment sets (Supplementary Table 4A). Parameters like CO_2_, H_2_, and CH_4_ correlated positively with CCA axis 1 and negatively with CCA axis 2. Positive correlation of axis 2 was observed with TIC, Fe, NO_3_^-^ and SO_4_^2−^, which on the other hand correlated negatively with axis 1. For BC enriched samples, CO_2_, H_2_, and CH_4_ correlated positively with both the axes (Supplementary Table 4B). Mn correlated positively with CCA axis 1 and negatively with CCA axis 2. Negative correlation effect of TIC and SO_4_^2−^ was observed for both the axes, whereas Fe and NO_3_^-^ correlated positively with CCA axis 2. The distribution of the microorganisms in the deeper rock (C4–C7) enrichments were strongly constrained by Mn, CO_2_, H_2_, and CH_4_ whereas the composition of the organisms in the shallower depth enrichment samples (C1–C3) could be guided by TIC, Fe, NO_3_^-^ and SO_4_^2−^ in both the enrichments. SIMPER analysis was done to determine the microbial taxa responsible for the differences between different depths in both enrichment conditions (Tables 3, 4). The topmost discriminating taxa were *Comamonas*, *Burkholderia-Caballeronia-Paraburkholderia*, *Klebsiella*, Enterobacter, Burkholderiaceae, Enterobacteriaceae and *Ralstonia*.

**Table 3:** SIMPER analysis of microbial communities from H_2_ + CO_2_ enrichments using rock samples from different depth.

Taxon	Average dissimilarity	Contribution %	Cumulative %
*Comamonas*	20.11	13.72	13.72
*Burkholderia-Caballeronia-Paraburkholderia*	16.64	11.3	25.02
*Klebsiella*	9.88	6.74	31.76
Enterobacteriaceae	7.73	5.24	37
*Enterobacter*	6.59	4.51	41.51
*Ralstonia*	6.62	4.42	45.93
Burkholderiaceae	7.63	4.14	50.07
*Pseudomonas*	0.88	2.42	52.49
Thaumarchaeota_Group1.1 c	0.79	2.1	54.59
*Methyloversatilis*	0.72	2.05	56.64
*Rhodanobacter*	1.00	1.77	58.41
*Cloacibacterium*	0.009	1.55	59.96
*Conexibacteria*	0.27	1.42	61.38
*Pseudarcicella*	0.38	1.13	62.51
*Corynebacterium 1*	0.41	0.93	63.44

**Table 4:** SIMPER analysis of microbial communities from HCO_3_^−^ enrichments using rock samples from different depth.

Taxon	Average dissimilarity	Contribution %	Cumulative %
*Comamonas*	0.18	13.18	13.18
*Burkholderia-Caballeronia-Paraburkholderia*	0.029	10.1	23.28
*Klebsiella*	0.009	6.05	29.33
Enterobacteriaceae	0.198	5.39	34.72
*Enterobacter*	0.009	4.81	39.53
Burkholderiaceae	0.833	4.62	44.15
*Ralstonia*	0.139	3.48	47.63
*Nocardioides*	4.77	2.93	50.56
*Aeromonas*	4.02	2.65	53.21
*Kurthia*	2.66	1.77	54.98
*Pseudomonas*	2.6	1.46	56.44
*Corynebacterium 1*	2.36	1.3	57.74
*Sphingomonas*	3.13	1.29	59.03
*Anaerolina*	1.71	1.12	60.15
JG30-KF-CM45	1.48	0.98	61.13

### Correlation among microbial phyla and co-occurrence network analysis

The Spearman correlation was used to identify the population at the phylum level that shared strong correlation amongst each other. In HC amended sets (Supplementary Figure 6), enriched members of the bacterial phyla Bacteroidetes, Spirochaetes, Cyanobacteria, Chloroflexi, WPS-2, and Patescibacteria as well as archaeal Euryarchaeota and Thaumarchaeota members constituted a strongly correlated clade. Armatimonadetes, Chlamydiae, Actinobacteria, Fusobacteria, Planctomycetes and Firmicutes populations formed the second guild. Coprothermobactereota, Altiarchaeota, Crenarchaeota, Caldiserica, Thermotogae, Verrucomicrobia constituted the third group. Lentisphaerae, Omnitraphechaeota, Synergistis and members belonging to FCPU426, Latescibacteria and Zixibacteria formed smaller mutually correlated clade. Proteobacteria, in spite of being the most abundant phylum, showed negative correlation with other enriched phyla. In the case of BC enrichments (Supplementary Figure 7) Chloroflexi, Gemmatimonadetes, Verrucomicrobia, Firmicutes, Actinobacteria and Bacteroidetes formed a strongly correlated group. The other two groups with strong correlation were formed by Cyanobacteria, Euryarchaeota, Deinicoccus Thermus and Crenarchaeota, Fibrobacteres, Tenericutes, respectively. Broadly, chemo/litho/autotrophic members of Cyanobacteria, Choloroflexi, Verrucomicrobia, Altiarchaeota were observed to correlate with members of Coprothermobactereota, Tenericutes and Gemmatimonadetes that are known to follow hetero/organotrophic lifestyle mostly with fermentative type of metabolism.

**Figure 7:**
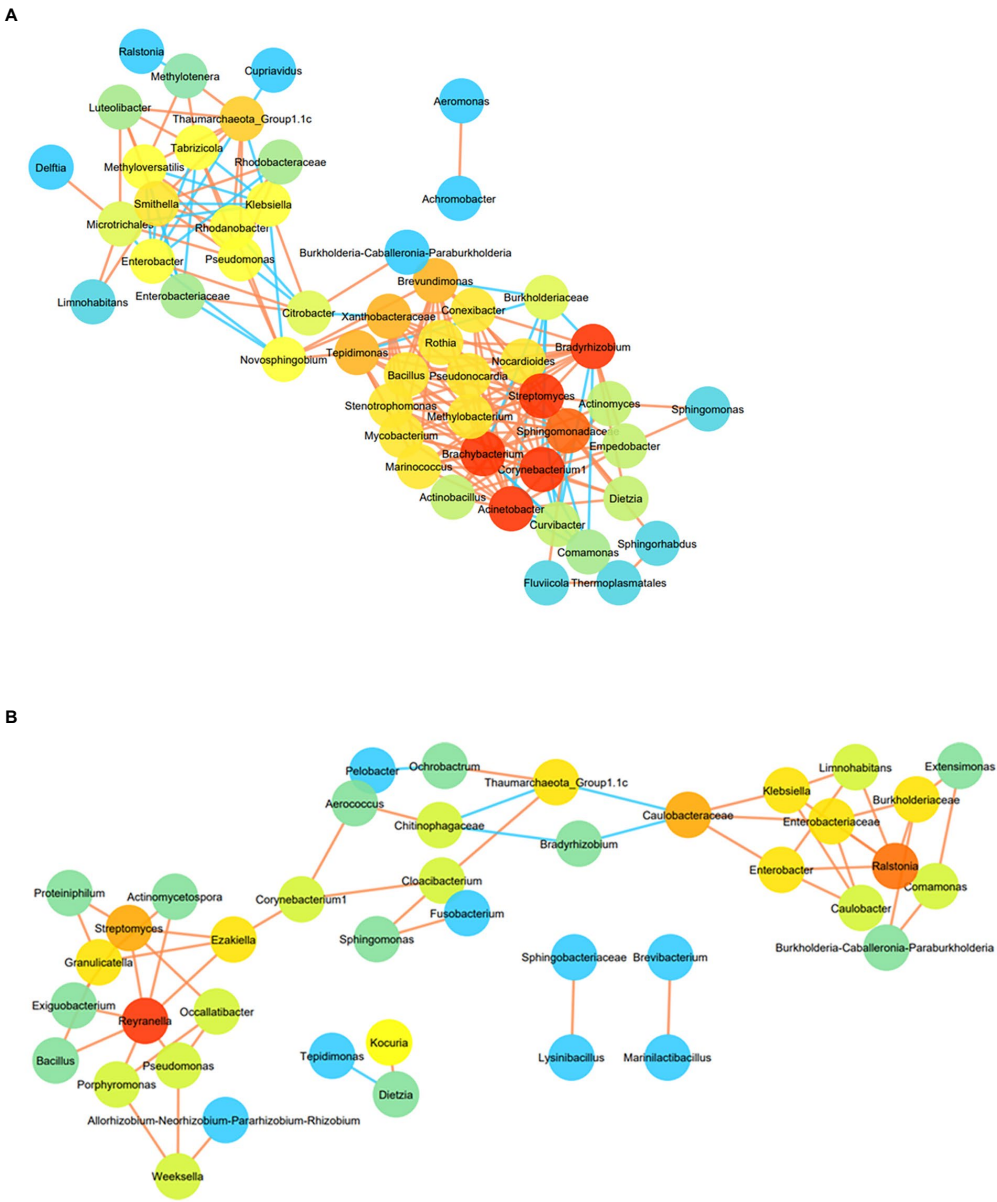
**(A)** Co-occurrence network of OTUs enriched from at least 4 samples (at the lowest taxa level) in HC enrichment. Node colour are proportional to the number of degrees. Nodes with higher degree are orange in colour than those with lower connections (blue in colour). Colour of the edges represents the strength of the Spearman’s correlations between the nodes. Edges with stronger correlation are orange in colour. For the Spearman correlation values |*r*| > 0.9 are only considered. **(B)** Co-occurrence network of OTUs enriched from at least 4 samples (at the lowest taxa level) in BC enrichment. Node colour are proportional to the number of degrees. Nodes with higher degree are orange in colour than those with lower connections (blue in colour). Colour of the edges represents the strength of the Spearman’s correlations between the nodes. Edges with stronger correlation are orange in colour. For the Spearman correlation values |*r*| > 0.9 are only considered.

The co-occurrence pattern of the H_2_ + CO_2_ or HCO_3_^−^ enriched microorganisms was analysed using network inferences based on strong and significant correlations, at the lowest taxonomic levels for OTUs present in at least four samples (Figures 7A,B). In HC enriched sets, the resulting microbial network consisted of 51 nodes and 187 edges (Figure 7A). The network consisted of two major hotspots (regions with highly connected taxa), with *Bradyrhizobium, Brachybacterium, Streptomyces, Acinetobacter, Corynebacterium 1*, being the taxa with the highest number of connections (16 each), followed by Sphingomonadaceae (15 connections). These taxa were found to be connected to *Conexibacter, Nocardiodes, Pseudonocardia, Methylobacterium, Marinococcus, Stenotrophomonas, Actinobacillus, Curvibacter, Comamonas* and Thermoplasmatales members. The second hotspot was formed by members belonging to Thaumarchaeota_Group 1.1c, *Methyloversatilis, Rhodanobacter, Pseudomonas*, Microtrichales, *Smithella, Ralstonia* and *Cupriavidus*. The most dominant taxa of the HC enrichments like *Comamonas, Burkholderia-Caballeronia-Paraburkholderia*, Enterobacteriaceae members (maximally dominant in C4–C7 enrichments) showed very few connections (mostly negative) with the other groups, hinting upon the fact that depth of the samples also played a role in determining the correlation amongst the enriched members. Members belonging to Burkholderiaceae, Chloroflexi, *Alicycliphylus*, *Brachymonas, Citrobacter, Burkholderia-Caballeronia-Paraburkholderia, Comamonas, Cupriavidus, Ralstonia* and *Hydrogenophaga* showed strong positive correlation with depth, and other geochemical parameters like CH_4_, Mn, H_2_, CO_2_ as depicted in Supplementary Figure 8A. Bacterial taxa like *Anoxybacillus, Conexibacter, Sideroxydans, Anaerococcus, Anaerobranca*, Chloroflexi_KD4-96, *Pseudomonas* as well as archaeal taxa belonging to Methanomassillicoccalles and *Methanosaeta* were observed to correlate positively with Fe, SO_4_^2−^ and NO_3_^−^, and negatively with depth and CH_4_, Mn, H_2_ (Supplementary Figure 8B).

Co-occurrence network for BC enriched samples were formed of three interconnected hotspots comprising of 41 nodes and 56 edges (Figure 7B). *Reyranella* was found to be the taxa with highest degree connected to seven other taxa nodes including *Exiguobacterium, Bacillus, Streptomyces, Actinomycetospora* and *Pseudomonas*. *Ralstonia* was found to act as the nucleus node of the second hotspot connecting to six other taxa namely, Enterobacteriaceae, Burkholderiaceae, *Klebsiella, Comamonas, Enterobacter* and *Limnohabitans*. Members belonging to Thaumarchaeota, *Aerococcus, Sphingomonas* and Chitinophagaceae formed the third hotspot. *Methanolinea,* Chroococcidiopsis, *Xanthobacter, Ralstonia, Cupriavidus, Curvibacter, Methylophilus*, Syntrophaceae and Burkholderiaceae members were found to be correlated positively with depth and parameters including CO_2_, H_2_, CH_4_ and Mn (Supplementary Figure 9A), whereas members of Thaumarchaeota_Group1.1c, Thermomicrobiales, Methylophilaceae, *Thermoactinomyces, Thiomonas, Sporosarcina* had strong positive correlation with SO_4_^2−^, Fe and NO_3_^-^ but was found to correlate negatively with depth, Mn, CH_4_ and H_2_ (Supplementary Figure 9B).

### Analysis of phylogenetic relationship among operational taxonomic units

To determine the evolutionary history and gain insight about the relationships of the reactivated organisms, Neighbour Joining (NJ) phylogenetic analysis of 15 most abundant OTUs and cyanobacterial OTUs from both inorganic enrichments (HC and BC, making a total of 36 query OTUs) were performed by constructing a bootstrap tree (Supplementary Figure 10). The presence and relative abundance of these OTUs were depicted in the form of heat map beside each OTU. OTUs observed under both conditions were designated in green, the ones enriched only in BC sets were designated in blue and the OTU that belongs only to HC enrichment set was marked in red. OTUs enriched under both conditions, belonging to bacterial members like *Burkholderia-Caballeronia-Paraburkholderia*, *Klebsiella*, *Rhodanobacter*, *Methyloversatilis*, *Pelomonas*, *Comamonas*, *Aeromonas*, *Kurthia*, *Nocardiodes*, *Corynebacterium 1*, *Dongia*, *Gemella, Deinococcus*, etc. and archaeal group Thaumarchaeota were observed to be phylogenetically connected to sequences retrieved from deep terrestrial subsurface sediments from USA, subsurface clay rock, sediment from Eyreville drill corehole in Chesapeake bay, deep sea, deep hydrothermal vent in Nankai Trough, Hadal Deep Biosphere Mariana Trench and hydrothermal aquifer in Naica Mines. OTU affiliated to *Conexibacter* (unique to HC enrichment) was found to be phylogenetically similar (with 1,000 bootstrap) to an endolithic bacterial sequence from serpentinite-hosted ophiolite massif of the Alpine-Apennine chain in Italy. Sequences belonging to *Fluviicola, Anaerolinea* and Prolixibacteraceae were enriched only under BC enrichment condition and were found to be similar to sequences from deep subsurfaces of sedimentary rock Japan, Paris basin aquifer and Mariana Trench. Cyanobacterial sequences enriched from both sets showed strong matches with other cyanobacterial sequences retrieved from granitic subsurface environment in Tokyo, Clay rock borehole water of Switzerland, deep subsurface of South Africa and Mariana Trench.

### Prediction of metabolic functionality *via* PICRUSt v2 analysis

**Figure 8:**
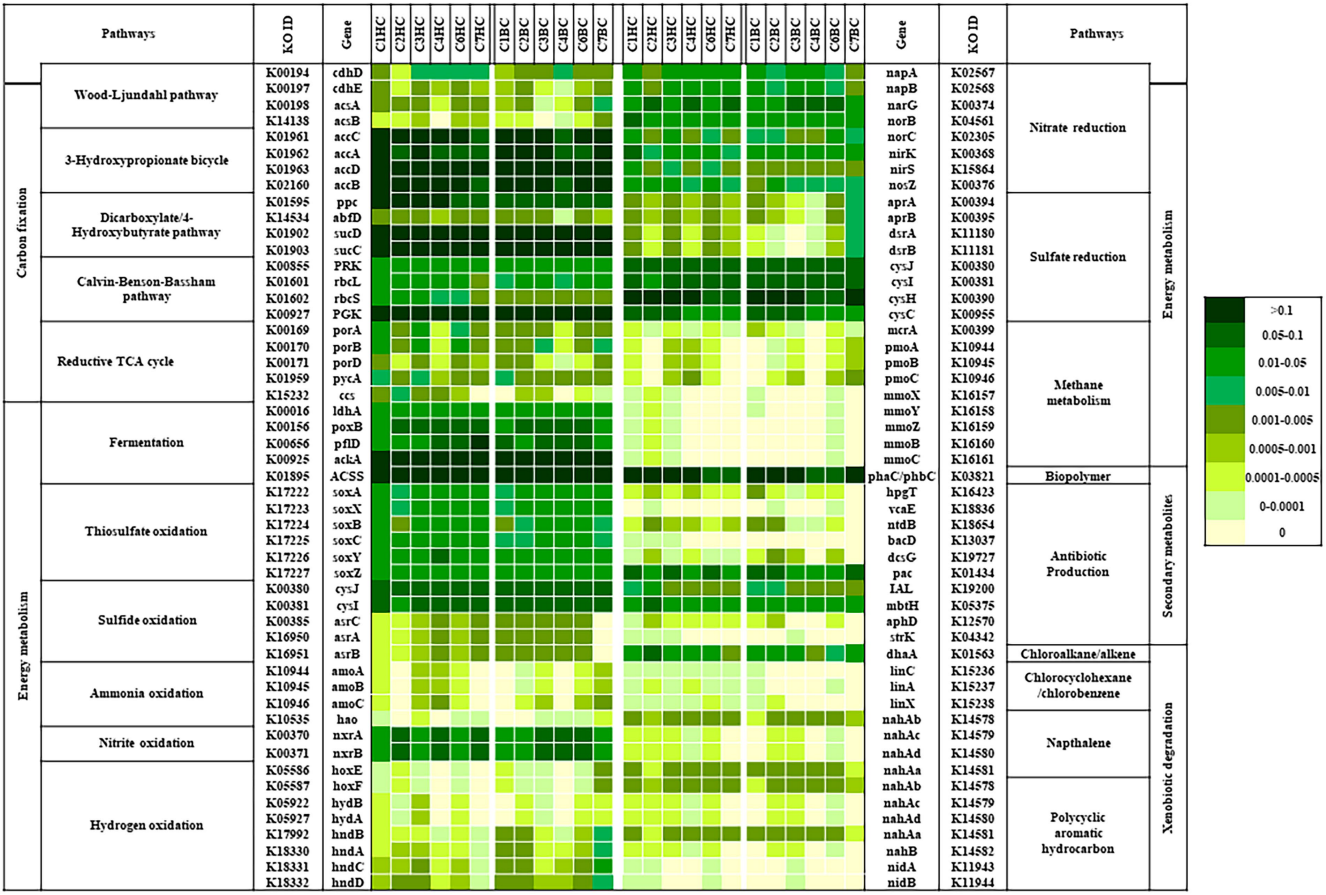
Relative abundance of predicted genes related to CO_2_ fixation pathway, lithotrophy related/energy metabolism and genes related to other important metabolism (amongst metabolic genes) as predicted by PICRUSt v2.

The bacterial community analysis performed in this study suggested the presence of diverse bacterial populations with varied predicted metabolic functions in deep terrestrial subsurface. PICRUSt v2 based predictive metabolic profiling was done to gain insights into the metabolic potential of such microorganisms that help them to sustain in a subsurface environment. The relative abundances of genes related to different metabolic traits, that were predicted from our enrichments have been presented in Figure 8. Analysis revealed that the genes known to be involved in carbon cycling pathways, mainly in carbon fixation *viz.* acetyl-CoA decarbonylase (*acs*ABCD), formylmethanofuran dehydrogenase (*fmd*ABCDE; genes for CO_2_ fixing WL pathway in acetogens and methanogens respectively), acetyl-CoA carboxylase, (*acc*ABCD; for HCO_3_^−^ fixing 3-HP bicycle), phosphoenolpyruvate carboxylase (*ppc*), 4-hydroxybutyryl-CoA dehydratase, (*abf*D; for DC/4HB cycle, CO_2_ and HCO_3_^−^ fixing), ribulose-bisphosphate carboxylase (*rbc*S/L), phosphoribulokinase (*prk*; for CO_2_ fixing CBB cycle) and Incomplete reductive TCA cycle, were predicted in all enrichment samples.

Lithotrophic metabolisms other than carbon dioxide and bicarbonate fixation play an important role in driving the energy cycle in the deep (Lovley and Chapelle, 1995; Osburn et al., 2014). The relative abundance of genes related to various lithotrophy related metabolism (as predicted) were presented in Figure 8, Energy metabolism.

Methane metabolism: Genes related to methane production (methyl-coenzyme M reductase; *mcrA*) and oxidation (methane monooxygenase; *pmo, mmo*) were predicted in most of the enrichment samples except in C7HC and C4BC. Overall relative abundance of genes *mcr*, *pmo* or *mmo* was found to be considerably lower than the abundance of other metabolic genes related to lithotrophy.

Nitrogen metabolism: The predicted presence and abundance of nitrogen metabolism genes in all enrichment sets hinted upon the significance of such metabolism. Genes related to nitrite oxidation (nitrate reductase / nitrite oxidoreductase; *nxr*), nitrate reduction (nitrate reductase; *nar*/*nap*, nitrite reductase; *nir,* nitric oxide reductase; *nor* and nitrous-oxide reductase; *nos*) and ammonia oxidation (ammonia monooxygenase; *amo,* hydroxylamine dehydrogenase; *hao*) were predicted in all enrichment sets.

Sulfur metabolism: Genes known to be involved in sulfide (anaerobic sulfite reductase; *asr, cys*) and thiosulfate (*sox*ABCXYZ) oxidation, sulfate reduction (adenylylsulfate reductase; *apr,* dissimilatory sulfite reductase; *dsr,* sulfite reductase; *cys*), were also predicted from the enriched samples. However, the predicted relative abundance of dissimilatory sulfate reduction genes was lower than genes involved in assimilatory sulfate reduction.

Hydrogen oxidizing genes (hydrogenases; *hnd, hyd, hox*) were predicted to be more abundant in the deeper rock sample enrichments. Genes for production of acetate, lactate and pyruvate (*ldh, pox, pfl, ack, ACSS*) were predicted in all the enrichment samples.

Presence of genes related to synthesis of secondary metabolites like polyhydroxyalkanoate, polyhydroxybutyrate (*pha/phb*), antibiotic (*vca, ntd, bac, dcs, pac, mbt, str*), as well as degradation of chloroalkane (*dha*), chlorocyclohexane (*lin*), naphthalene (*nah*) and polycyclic aromatic hydrocarbon (*nah, nid*) could also be predicted from both the enrichment sets (Figure 8; Secondary metabolites and Xenobiotic degradation).

## Discussion

In spite of extreme high temperatures, lithostatic pressure, increasing dryness and aridity, decreasing porosity, scarcity of survival space and nutrient deprivation, deep terrestrial subsurface environments have been found to be inhabited by diverse microbial communities that have been shaped by the prevailing physical and geochemical factors (Nyyssonen et al., 2014; Kieft, 2016; Dutta et al., 2018, 2019; Sahu et al., 2022). The life in deep biosphere is considered to be driven by geologically produced CO_2_, H_2_ and/or reduced S and N driven chemoautotrophic production of small organic molecules which can be utilized further as nutrient resources. (Purkamo et al., 2015; Adhikari et al., 2016). In the present study, we have provided chemolithoautotrophic conditions, *viz.*, two different inorganic carbon sources (CO_2_/HCO_3_-), H_2_ / NH_4_^+^ as electron donors and NO_3_^−^ and/or SO_4_^2−^ as electron acceptors, to the deep subsurface rock hosted communities to enrich chemolithotrophic microorganisms. It has resulted in successful enrichment of diverse groups of microorganisms (Bacteria and Archaea) as evident from the outcome of the growth measurement experiments (Figure 1) and formation of different metabolites like lactate, formate and acetate. In taxon-based approaches, accurate assessment of species richness is considered as it is suggested to be necessary for effective analysis of the bacterial community (Kim et al., 2017). The diversity indices have suggested variable levels of diversity and richness in both HC and BC amended incubations. The outputs of Chao1 and ACE (richness estimators) indices mark the enrichment sets as microbially rich which is also supported by the Shannon diversity index values and corroborated well with the previous observations, on granitic samples belonging to KSZ (Dutta et al., 2018). The contrast between increase of gene copy numbers (on average) and the decrease in the number of OTUs (in deeper rock enrichments) has suggested that specific microbial taxa members dominated the enrichments from deeper rocks that also supported our taxonomic data where fewer groups made up the enriched communities of deeper rocks. On the other hand, enrichment cultures of the relatively shallower rocks exhibited more diverse organisms. The high percentage of unique OTUs in each enrichment set (Supplementary Figures 2A,B) possibly indicated that the community composition of the rock enrichment cultures were significantly shaped by the intrinsic nature of the rock samples obtained from different depths. 16S rRNA gene sequencing indicated the ubiquitous predominance of phylum Proteobacteria, (Gamma-, Alpha-, Delta-, Betaproteobacteriales), with increasing abundance in the deeper rock enrichment samples (Supplementary Figure 3). Several members of Proteobacteria are reported to follow autotrophic mode of nutrition along with H_2_/Fe^2+^/S oxidizing potential (Nakagawa and Takai, 2008; Brazelton et al., 2012; Purkamo et al., 2015; Kumar et al., 2018). Some of the members were reported to be capable of dark bicarbonate assimilation (Alonso-Saez et al., 2010). However, plenty of proteobacterial taxa follow heterotrophic mode of nutrition and it might be a possibility that these heterotrophic members survive on the organic metabolic byproducts of other autotrophic organisms. Actinobacteria, Firmicutes, Bacteroidetes, Acidobacteria are among the other dominant phyla found to be enriched in this study. Presence of Actinobacteria in the enrichment samples has corroborated well with extreme stress-tolerant ability (including high temperature and salinity) of its members as well as previous reports on their abundance within the igneous crust underneath the Deccan Traps (Idris et al., 2017; Dutta et al., 2018; Bull and Goodfellow, 2019; Hui et al., 2021; Sahu et al., 2022). Members of Firmicutes are found to be capable of extracting energy and other nutrients from diverse inorganic resources and also assimilate CO_2_ (Salehizadeh et al., 2020). Acidobacterial members are reported to harbour a versatile repertoire of genes necessary to use ammonia and/or nitrate/nitrite as their N sources and H_2_ at atmospheric concentrations (Eichorst et al., 2018). Thermophilic, anaerobic, chemolithoautotrophic Chloroflexi members are described to be environmentally widespread and reported to harbour genes involved in adaptation against various environmental stress conditions (Wasmund et al., 2014; Kadnikov et al., 2019; Vuillemin et al., 2020). They are reported to contain potential for fermentation, CO_2_ fixation and acetogenesis *via* the Wood–Ljungdahl (WL) pathway (Vuillemin et al., 2020). Presence of Thaumarchaeota and Euryarchaeota (known to harbor thermophilic ammonia oxidizers and methanogens, respectively) in the enrichments probably hinted about their chemolithoautotrophic potential that helped them to thrive within this extremely energy-limited terrestrial subsurface environments while playing a prominent biogeochemical role (Seán et al., 2003; Ragon et al., 2013; Cardarelli et al., 2020). The enriched taxa that are found capable of adaptation to diverse environmental stresses, CO_2_ fixation, and lithotrophic metabolisms could possibly be attributed and linked to the existing environmental and geochemical conditions of the KSZ deep subsurface (Dutta et al., 2018, 2019; Sahu et al., 2022). The rocks in this dark biosphere have not seen sunlight since the geologic past (Dutta et al., 2018), yet interestingly, enrichment of Cyanobacteria (thought to require sunlight to survive) were observed in HC and BC (minute fraction) enrichment sets. Previous studies have shown presence of Cyanobacteria in igneous crust (basalt and granitic rocks) from deep subsurface of the Deccan traps (Dutta et al., 2018). Members of Cyanobacteria have been reported to be ecologically versatile and to be associated with a potential for H_2_-based lithoautotrophic metabolism in deep terrestrial subsurface rocks (Momper et al., 2017; Kadnikov et al., 2018; Puente-Sánchez et al., 2018). Considering the recent report on presence of H_2_ in the deep subsurface of Koyna region (Podugu et al., 2019), the presence of Cyanobacterial members (mainly in HC enrichments) could probably be linked to their H_2_-based lithoautotrophic metabolic ability. Previous investigations suggested that Cyanobacterial members can also switch to fermentative lifestyle when required (Dutta et al., 2018 and references therein).

NMDS analyses (Figure 5) has illustrated the community assemblages and the role of subsurface geochemistry (including depth and different enrichment substrates) in shaping the microbial community. The role of geochemical factors on microbial community structure and function in various crystalline deep biospheres have previously been described (Brazelton et al., 2013; Magnabosco et al., 2014). In the present study, depth is found to be the influential environmental variable which significantly affected the microbial community structure. Even though substrate has an influence on microbial composition in enrichment sets, it is subtle compared to the stark effect of depth of the different rocks used for enrichments. Depth wise distribution of the enrichment samples irrespective of the enrichment substrates is also observed in the CCA analysis (Figures 6A,B) along with strong constraint of Mn, CO_2_, H_2_, and CH_4_ in deeper rock (C4–C7) enrichments, and TIC, Fe, NO_3_^−^ and SO_4_^2−^ in the shallower rock (C1–C3) enrichments. The results of SIMPER analysis has indicated that facultatively lithotrophic CO or H_2_-oxidizing, NO_3_^−^reducing and the most abundant *Comamonas* (Willems and De Vos, 2006) to be the maximum contributor in depth dependent average dissimilarity.

Correlation among the enriched microbial phyla based on their abundance highlighted the possible interplay among the enriched groups and a marked effect of depth. The groups constituting each of the strongly correlated clades has shown variety in their abundances with the change in the sample depth, either following a pattern or predominating at some specific depths. This difference in abundances could be attributed to the existing environmental conditions of that sample which may have favoured the growth of specific groups. Syntropy has been reported to play an important role in deep subsurface environments (Matsushita et al., 2020). Autotrophic taxa proficient of C fixation (e.g., Cyanobacteria), also capable H_2_-based metabolism (Puente-Sánchez et al., 2018), Chloroflexi, and NH_4_ oxidizing/ methanogenic archaeal groups are observed to associate with organotrophic acetate assimilating Bacteroidetes (Kadnikov et al., 2020), fermentative Firmicutes and Fusobacteria members (Rogers et al., 1998; Gupta et al., 2018).

Microbial co-occurrence analysis has indicated the presence of distinct and well-defined networks across the enriched samples (Figures 7A,B), which corroborated well with the correlation-based observation of association between autotrophic and heterotrophic communities. Taxa affiliated to the first hotspot have previously been reported to be involved in diverse, yet deep biosphere relevant metabolic processes. Organisms belonging to *Bradyrhizobium, Brachybacterium, Streptomyces, Acinetobacter, Conexibacter, Pseudonocardia, Curvibacter,* and *Comamonas* have been known to tolerate enhanced CO_2_ level or utilize H_2_ and/or CO/CO_2_ as sole energy and carbon source (Willems and De Vos, 2006; Greening et al., 2018; Takors et al., 2018; Gülay et al., 2018; Islam et al., 2020). These organisms are observed to correlate strongly amongst each other and with organotrophic *Nocardiodes*, methylotrophic *Methylobacterium* and mixotrophic Thermoplasmatales members (Huber and Stetter, 2006; Yoon and Park, 2006). The second hotspot depicted strong connections between lithotrophic organisms like ammonia oxidizing, dark carbon fixing Thaumarchaeota_ Group1.1c, SO_4_^2−^, CO and H_2_ utilizing Microtrichales, lithoautotrophic Rhodobacteraceae members, methylotrophic *Methyloversatilis* and metabolically versatile *Pseudomonas* members (Ghosh and Dam, 2009; Lapidus et al., 2011; Pohlner et al., 2019; Cardarelli et al., 2020). The network of HCO_3_- enrichments depicted the presence of smaller interconnected subnetworks wherein dominant taxa of deeper rock enrichments (*Ralstonia, Comamonas*, *Burkholderia-Caballeronia-Paraburkholderia*, Burkholderiaceae etc.), and the dominant taxa of shallower rock enrichments (*Sphingomonas, Nocardiodes, Corynebacterium 1, Aerococcus* etc.) formed two hotspots. The interconnected taxa in these hotspots were reported to follow chemolithoautotrophy or organotrophy. The third hotspot mainly consisted of highly connected organotrophic and metabolically versatile taxa members (Yoon and Park, 2006; Cardarelli et al., 2020).

Overall, these correlation studies and co-occurrence networks distinctly highlighted the strong mixotrophic interconnection and association between enriched lithoautotrophic and organotrophic organisms, where, based on their preferred modes of nutrition they help in the successful utilization of the available substrates and drive the energy requirement of the enriched community. Similar associations amongst autotrophic and heterotrophic populations have been reported in previous studies on the deep subsurface settings and igneous rock environments (Hallbeck and Pedersen, 2012; Pedersen, 2013; Purkamo et al., 2015; Adhikari et al., 2016; Wu et al., 2016; Momper et al., 2017; Dutta et al., 2018; Sahu et al., 2022). A possible reason for this association can be the utilization of organic by products (exudated by chemolithoautotrops) by the interacting heterotrophic community (Wu et al., 2016).

The observation on the correlation networks of the enriched taxa with depth and other geochemical parameters corroborated well with similar reports from other deep subsurface environments with dominant chemolithotrophic mode of nutrition (Sahl et al., 2008; Yoon et al., 2008; Cramm, 2009; Panda et al., 2009; Purkamo et al., 2015; Carr et al., 2018; Gülay et al., 2018; Hädrich et al., 2019; Cardarelli et al., 2020; Nangle et al., 2020; Jakus et al., 2021). Members of *Burkholderia-Caballeronia-Paraburkholderia* (one of the major taxa enriched from our study and having positive correlation with sample’ depth) have also shown highest correlation with depth and connections with geochemical parameters like H_2_ and CH_4_ in an earlier study related to the rock metagenome (Sahu et al., 2022).

A similarity search of the most abundant OTUs revealed a significant relationship with already reported species from similar dark, aphotic and extremophilic deep subsurface habitats with chemoauto/organotrophic metabolic characteristics (Brown and Balkwill, 2009; Ragon et al., 2013; Ino et al., 2018; Supplementary Figure 10). These significant matches revealed that the groups enriched from our study might follow similar chemolithoautotrophic nutritional modes as reported previously. Considering anaerobic environmental conditions, SO_4_^2−^, NO_3_^−^, Mn^4+^, Fe^3+^ and CO_2_, could be used as the most “auspicious” (Purkamo et al., 2015) terminal electron acceptors, and organisms metabolising the same, could thrive and play a dominant role in these enrichment samples.

Understanding the dynamics of processes carried out by ubiquitous microorganisms of these deep terrestrial systems has been important as it holds broad significance for the global biogeochemical cycles (Hernsdorf et al., 2017). Investigation of the 16S rRNA gene amplicon from the total community DNA of the enrichment cultures for function prediction elucidated the metabolic capacities of the cultured communities, especially in terms of their carbon and energy metabolism processes. The prediction of major metabolic pathways gave us an insight of the metabolic repertoire present in these enrichment cultures (Figure 8).

CO_2_ assimilation: Chemically stable and unreactive CO_2_ must be reduced to enable its incorporation into biological molecules. In the recent past, CO_2_ fixation pathways used by autotrophic microorganisms have received interest for biotechnological applications, since it could provide biological routes for *de novo* generation of fuels and small organic molecules (Hawkins et al., 2013). Analysis of the metabolic repertoire of the samples predicted the prevalence of mixotrophic (both autotrophic and organotrophic) modes of C-assimilation. 3HP bicycle, DC/4HB cycle, CBB and Wood-Ljungdahl pathways are predicted to be the most significant autotrophic C assimilation processes. The WL pathway have been described as the most energy inexpensive pathway with net synthesis of ATP along with carbon fixation (Berg, 2011; Liu et al., 2020). Prediction of the WL pathway key genes in the enriched microbiome could be justified owing to its suitability as a favoured C fixation processes in these extreme energy deprived habitats (Momper et al., 2017). Bioavailability of surface-derived organic C has been found to be limited in the terrestrial granitic subsurface and hence many resident heterotrophs rely on *in-situ* production of fixed carbon by chemoautotrophs. Key genes for 3-HP pathway and DC/4HB pathway are predicted from the cultures. These pathways have been known to assimilate bicarbonate *via* acetyl-CoA/propionyl-CoA carboxylase/ PEP carboxylase respectively, and produce pyruvate and acetyl CoA (Liu et al., 2020). Both cycles have very high energy requirements, and the reason these cycles have survived through evolution might be due to their oxygen tolerance and bicarbonate assimilation instead of CO_2_ (Liu et al., 2020). The latter is advantageous, as the intracellular bicarbonate concentration can be much higher than the intracellular CO_2_ concentration. Putative presence of the key gene Acetyl-CoA carboxylase (*acc*) also suggested the possibility of dark C assimilation in the deep subsurface by the enriched community (Purkamo et al., 2015). This enzyme converts Acetyl-CoA to Malonyl-CoA, which can be used in diverse biosynthetic pathways (e.g., fatty acid biosynthesis and secondary metabolism, Momper et al., 2017). The DC/4HB cycle has been known to fix one mole of CO_2_ *via* pyruvate synthase and one mole of bicarbonate *via* phosphoenolpyruvate (PEP) carboxylase, in anaerobes, as well as in facultative aerobes (Berg et al., 2010; Liu et al., 2020; Saini et al., 2011). The putative presence of genes related to this pathway might provide an added advantage of fixing both CO_2_ and bicarbonate *via* the same thereby managing the energy expenditure in these enriched communities. The energy requirement in this pathway is found to be lower than the other two bicarbonate fixing pathways (Berg et al., 2010; Liu et al., 2020). Organisms enriched in our samples, belonging to taxa like Chloroflexi-KD4-96, Microtrichales, Altiarchaeia, *Methanolinea*, *Ralstonia, Rhodobacteria, Acidithiobacillus, Conexibacter, Hydrogenophaga, Methanobacterium* etc., have been reported earlier to be capable of autotrophic fixation of CO_2_ (Beulig et al., 2015; Liu et al., 2020; Salehizadeh et al., 2020).

Lithotrophy related energy metabolism: Enriched endolithic microbial communities inhabiting rocks might carry out *in situ* primary metabolite production (Lovley and Chapelle, 1995; Stevens, 1997). Available evidence suggested the use of different chemolithoautotrophic mechanisms for exploiting this energy in the deep subsurface, such as S, N, Fe, Mn metabolism, H_2_ oxidation, CH_4_ metabolism and acetogenesis (Stevens, 1997; Osburn et al., 2014). The analysis of 16S rRNA gene amplicon inventory predicted the presence of genes for S (*aps, dsr, cys*, *asr, sox*), N (*nar, nir, nif, nap, nos,*), CH_4_ (*pmo, mmo, mcr*) metabolism, NH_4_ oxidation (*amo, hao*) and acetogenesis (WL pathway) in our enrichment cultures. The predictive abundance of SO_4_^2−^ reducing genes in the H_2_- induced enrichments hinted upon the fact that sulfate reduction dependent H_2_ oxidation, might be an ongoing process in the deeper rock enrichments (Pedersen, 2013). Predictive presence of H_2_ oxidizing genes (*hnd, hyd, hox*), mostly in the deeper sample enrichments, and the enrichment of members belonging to *Hydrogenophaga, Methanolinea, Ralstonia, Methanobacterium, Cupriavidus* and *Dehalococcidia* (all being previously reported as hydrogenotrophic organisms and positively correlated to depth in our study), indicated the value of H_2_ as an energy source and suggested that the enriched microbes could be involved in its utilization (Adhikari et al., 2016; Hernsdorf et al., 2017; Nangle et al., 2020; Yang et al., 2020). The enriched organisms (Thaumarchaeota_ Group1.1c, *Thermodesulfovibrio*, and *Burkholderia-Caballeronia-Paraburkholderia, Thiomonas*) are predicted to be capable of utilizing inorganic compounds to harbour energy for their sustenance where reduced S and N, could act as potent electron donors and SO_4_^2−^, NO_3_^−^, Mn^4+^ as electron acceptors to fuel the metabolism of the enriched community (Panda et al., 2009; Cardarelli et al., 2020). The predicted ability of the enriched taxa to carry out lithotrophic metabolisms could possibly be attributed and linked to the presence of the mentioned electron donors and acceptors in the KSZ deep subsurface environments (Dutta et al., 2018, 2019; Sahu et al., 2022). Putative presence of fermentative pathways for acetate (*pox, ACSS, ack*), lactate (*ldh*), pyruvate (*pfl*), and formate production could be an indication of organotrophy (Dutta et al., 2018), thereby corroborating with earlier reports of interplay between autotrophic and heterotrophic enriched community.

Other associated metabolisms (secondary metabolite, antibiotic production and xenobiotic degradation): Secondary metabolites resulting from biosynthetic reactions have tremendous potential to be novel agents performing as chemotherapeutics, nutraceuticals or other value-added products. The deep biosphere has been investigated for bioactive natural products, unique polysaccharides, lipids and enzymes (Pettit, 2011). Genes related to synthesis of different antimicrobial and medicinal compounds, biodegradable polymers and xenobiotic degradation were predicted from the 16S rRNA gene amplicon inventory of our study. This prediction could be linked to the enrichment of taxa like *Actinobacteria, Pseudomonas, Aeromonas, Alcaligenes, Ralstonia* and *Marinobacter*, few of which were reported earlier to be capable of production natural biodegradable polyesters (PHA, PHBs), antimicrobial compounds and degradation of xenobiotic compounds (Mullis et al., 2019; Moradali and Rehm, 2020; Miglani et al., 2022).

In conclusion, this study provided a detailed overview of indigenous microorganisms enriched from granitic rock core samples, drilled out from the reservoir triggered seismogenic zone in Koyna, Maharashtra, India. Our enrichment-based work showed that the deep, hot, crystalline granitic rocks host live microorganisms (Bacteria and Archaea) and these can be brought to culture using chemolithotrophic, anaerobic conditions, with inorganic carbon substrates like CO_2_ (along with H_2_ as electron donor) or HCO_3_^−^ (along with NH_4_ as electron donor) as the sole C source. Furthermore, the taxonomic compositional analyses indicated a statistically significant, distinct depth-wide partitioning of diverse chemolithotrophic Bacteria and Archaea enriched from these rocks. 16S rRNA gene-based community composition showed Proteobacterial members to be the dominantly enriched bacterial group with their abundance being higher in the deeper rock sample enrichments. Based on the results of predictive metabolic profiling, it can be assumed that assimilation of carbon with utilization of sulfur, hydrogen and nitrogen compounds coupled with fermentation and anaerobic respiration could act as the main energy drivers.

## Data availability statement

The datasets presented in this study can be found in online repositories. The names of the repository/repositories and accession number(s) can be found in the article/Supplementary material.

## Author contributions

SK and PS conceived the study, designed the experiments, arranged all the resources, prepared the manuscript and mentored the study. SM carried out the microcosm setups, microbiological analysis, statistical analysis, data analysis, and manuscript preparation. HB assisted in sampling, sub-coring of rocks and carrying out some experiments. KR carried out the statistical analysis. RS assisted in qPCR and sequence analysis. AS performed the sequencing. All authors contributed to the article and approved the submitted version.

## Funding

This study was supported by the Ministry of Earth Sciences (MoES), Government of India (project ID: MoES/P.O. (Seismo)/1(288)/2016 dated 16 March 2017).

## Conflict of interest

The authors declare that the research was conducted in the absence of any commercial or financial relationships that could be construed as a potential conflict of interest.

## Publisher’s note

All claims expressed in this article are solely those of the authors and do not necessarily represent those of their affiliated organizations, or those of the publisher, the editors and the reviewers. Any product that may be evaluated in this article, or claim that may be made by its manufacturer, is not guaranteed or endorsed by the publisher.

## Footnotes

^1^ www.arb-silva.de/documentation/release-132

^2^ http://www.genome.jp/kegg/

## Supplementary material

The Supplementary material for this article can be found online at:

https://www.frontiersin.org/articles/10.3389/fmicb.2022.1018940/full#supplementary-material

Supplementary Figure 1 OTU overlap within HC and BC enrichments to determine the unique and shared OTUs.

Supplementary Figure 2 OTU overlap within all **(A)**HC and **(B)**BC enrichments.

Supplementary Figure 3 Taxonomic distribution of the microbial communities in HC and BC enrichments at the phylum/class/order level.

Supplementary Figure 4 Heatmap displaying the relative abundance of less abundant taxa (abundance <0. 1%) detected in **(A)** HC and **(B)** BC enrichments.

Supplementary Figure 5 Rank abundance (based on relative percentage abundance) of less abundant OTUs (abundance <0. 1%) detected in **(A)** HC and **(B)** BC enrichments.

Supplementary Figure 6 Heatmap of the Spearman correlation based on abundance of all microbial phyla detected in HC enrichment (abundance values are mentioned in the first bracket).

Supplementary Figure 7 Heatmap of the Spearman correlation based on abundance of all microbial phyla detected in BC enrichment (abundance values are mentioned in the first bracket).

Supplementary Figure 8A Co-occurrence network of microbial communities (taxa level) in HC enrichment positively correlated with sample’s depth. Node colour are proportional to the number of degrees. Nodes with higher degree are orange in colour than those with lower connections. Colour of the edges represents the strength of the Spearman’s correlations between the nodes. Edges with stronger correlation are orange in colour. *Burkholderia-Caballeronia-Paraburkholderia* is designated as Bur-Cab-Para.

Supplementary Figure 8B Co-occurrence network of microbial community (taxa level) in HC enrichment negatively correlated with sample’s depth. Node colour are proportional to the number of degrees. Nodes with higher degree are green in colour than those with lower connections. Colour of the edges represents the strength of the Spearman’s correlations between the nodes. Edges with stronger correlation are green in colour.

Supplementary Figure 9A Co-occurrence network of microbial community (taxa level) in BC enrichment positively correlated with sample’s depth. Node colour are proportional to the number of degrees. Nodes with higher degree are orange in colour than those with lower connections. Colour of the edges represents the strength of the Spearman’s correlations between the nodes. Edges with stronger correlation are orange in colour. *Burkholderia-Caballeronia-Paraburkholderia* is designated as Bur-Cab-Para.

Supplementary Figure 9B Co-occurrence network of microbial community (taxa level) in BC enrichment negatively correlated with sample’s depth. Node colour are proportional to the number of degrees. Nodes with higher degree are green in colour than those with lower connections. Colour of the edges represents the strength of the Spearman’s correlations between the nodes. Edges with stronger correlation are green in colour.

Supplementary Figure 10 Neighbour joining tree based on top 30 OTUs and cyanobacterial OTU sequences and their related organisms showing phylogenetic relationship. Numbers at the nodes represent the bootstrap value (1,000 replicates). The colour codes depict the presence and abundance of OTUs in sample zones. S-Shallower rock enrichments (average of C1–C3), D- Deeper rock enrichments (average of C4–C7), HC- sets amended with H_2_ + CO_2_, BC- sets amended with Bicarbonate.
