# Supplementary material for: Depth wide distribution and metabolic potential of chemolithoautotrophic microorganisms reactivated from deep continental granitic crust underneath the Deccan Traps at Koyna, India
Source: Front Microbiol. 2022 Nov 24;13:1018940. doi: 10.3389/fmicb.2022.1018940 (PMC9731672; doi:10.3389/fmicb.2022.1018940)
Supplement: Supplementary Figure 1 — OTU overlap within HC and BC enrichments to determine the unique and shared OTUs. [file Data_Sheet_2.ZIP › Supp table 1, 2.pptx]

## Slide 1
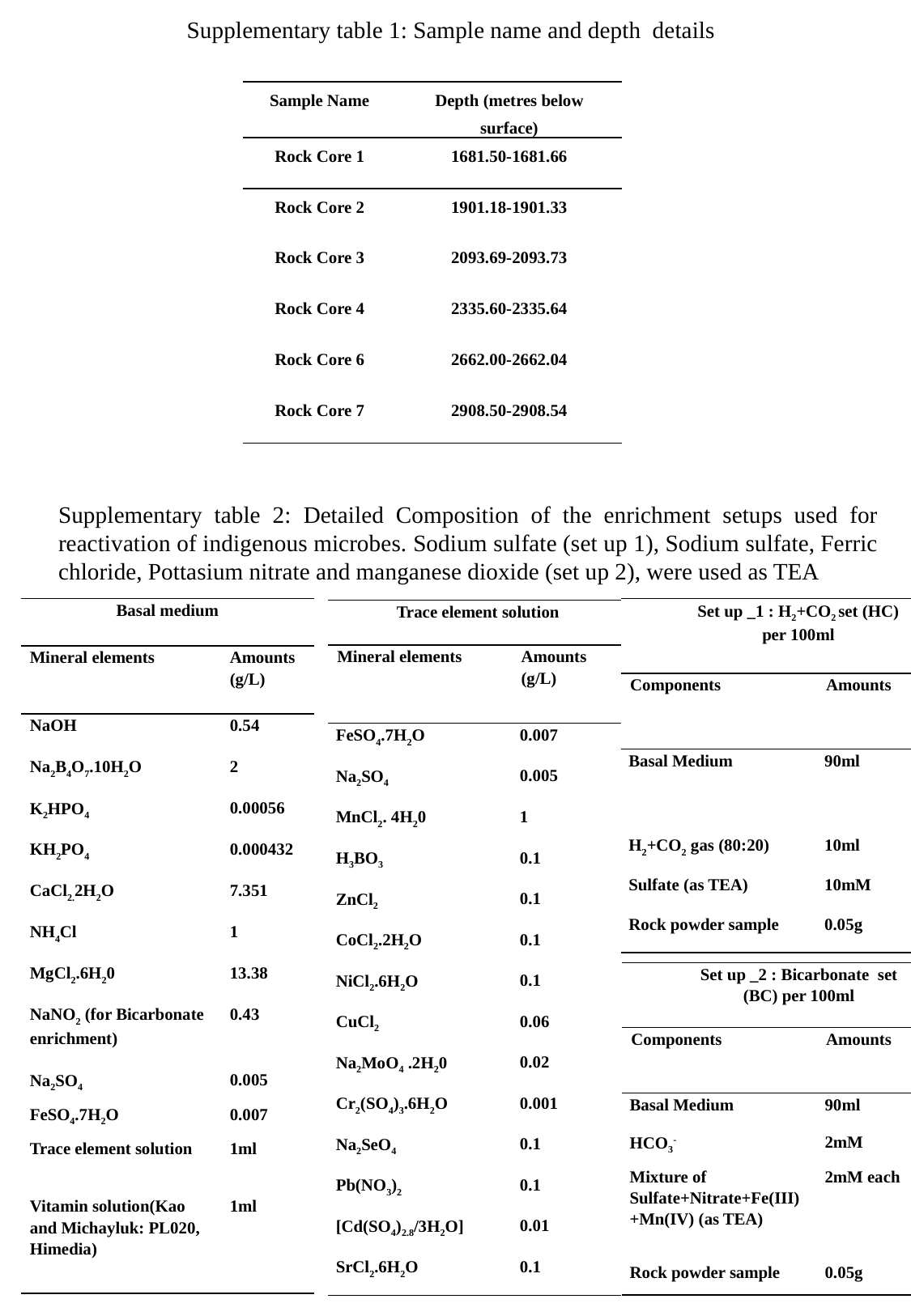

Supplementary table 1: Sample name and depth details
| Sample Name | Depth (metres below surface) |
| --- | --- |
| Rock Core 1 | 1681.50-1681.66 |
| Rock Core 2 | 1901.18-1901.33 |
| Rock Core 3 | 2093.69-2093.73 |
| Rock Core 4 | 2335.60-2335.64 |
| Rock Core 6 | 2662.00-2662.04 |
| Rock Core 7 | 2908.50-2908.54 |
Supplementary table 2: Detailed Composition of the enrichment setups used for reactivation of indigenous microbes. Sodium sulfate (set up 1), Sodium sulfate, Ferric chloride, Pottasium nitrate and manganese dioxide (set up 2), were used as TEA
| Basal medium | |
| --- | --- |
| Mineral elements | Amounts (g/L) |
| NaOH | 0.54 |
| Na2B4O7.10H2O | 2 |
| K2HPO4 | 0.00056 |
| KH2PO4 | 0.000432 |
| CaCl2.2H2O | 7.351 |
| NH4Cl | 1 |
| MgCl2.6H20 | 13.38 |
| NaNO2 (for Bicarbonate enrichment) | 0.43 |
| Na2SO4 | 0.005 |
| FeSO4.7H2O | 0.007 |
| Trace element solution | 1ml |
| Vitamin solution(Kao and Michayluk: PL020, Himedia) | 1ml |
| Set up \_1 : H2+CO2 set (HC) per 100ml | |
| --- | --- |
| Components | Amounts |
| Basal Medium | 90ml |
| H2+CO2 gas (80:20) | 10ml |
| Sulfate (as TEA) | 10mM |
| Rock powder sample | 0.05g |
| Trace element solution | |
| --- | --- |
| Mineral elements | Amounts (g/L) |
| FeSO4.7H2O | 0.007 |
| Na2SO4 | 0.005 |
| MnCl2. 4H20 | 1 |
| H3BO3 | 0.1 |
| ZnCl2 | 0.1 |
| CoCl2.2H2O | 0.1 |
| NiCl2.6H2O | 0.1 |
| CuCl2 | 0.06 |
| Na2MoO4 .2H20 | 0.02 |
| Cr2(SO4)3.6H2O | 0.001 |
| Na2SeO4 | 0.1 |
| Pb(NO3)2 | 0.1 |
| [Cd(SO4)2.8/3H2O] | 0.01 |
| SrCl2.6H2O | 0.1 |
| Set up \_2 : Bicarbonate set (BC) per 100ml | |
| --- | --- |
| Components | Amounts |
| Basal Medium | 90ml |
| HCO3- | 2mM |
| Mixture of Sulfate+Nitrate+Fe(III)+Mn(IV) (as TEA) | 2mM each |
| Rock powder sample | 0.05g |
