# Supplementary material for: Depth wide distribution and metabolic potential of chemolithoautotrophic microorganisms reactivated from deep continental granitic crust underneath the Deccan Traps at Koyna, India
Source: Front Microbiol. 2022 Nov 24;13:1018940. doi: 10.3389/fmicb.2022.1018940 (PMC9731672; doi:10.3389/fmicb.2022.1018940)
Supplement: Supplementary Figure 1 — OTU overlap within HC and BC enrichments to determine the unique and shared OTUs. [file Data_Sheet_2.ZIP › Supp table 3.pptx]

## Slide 1
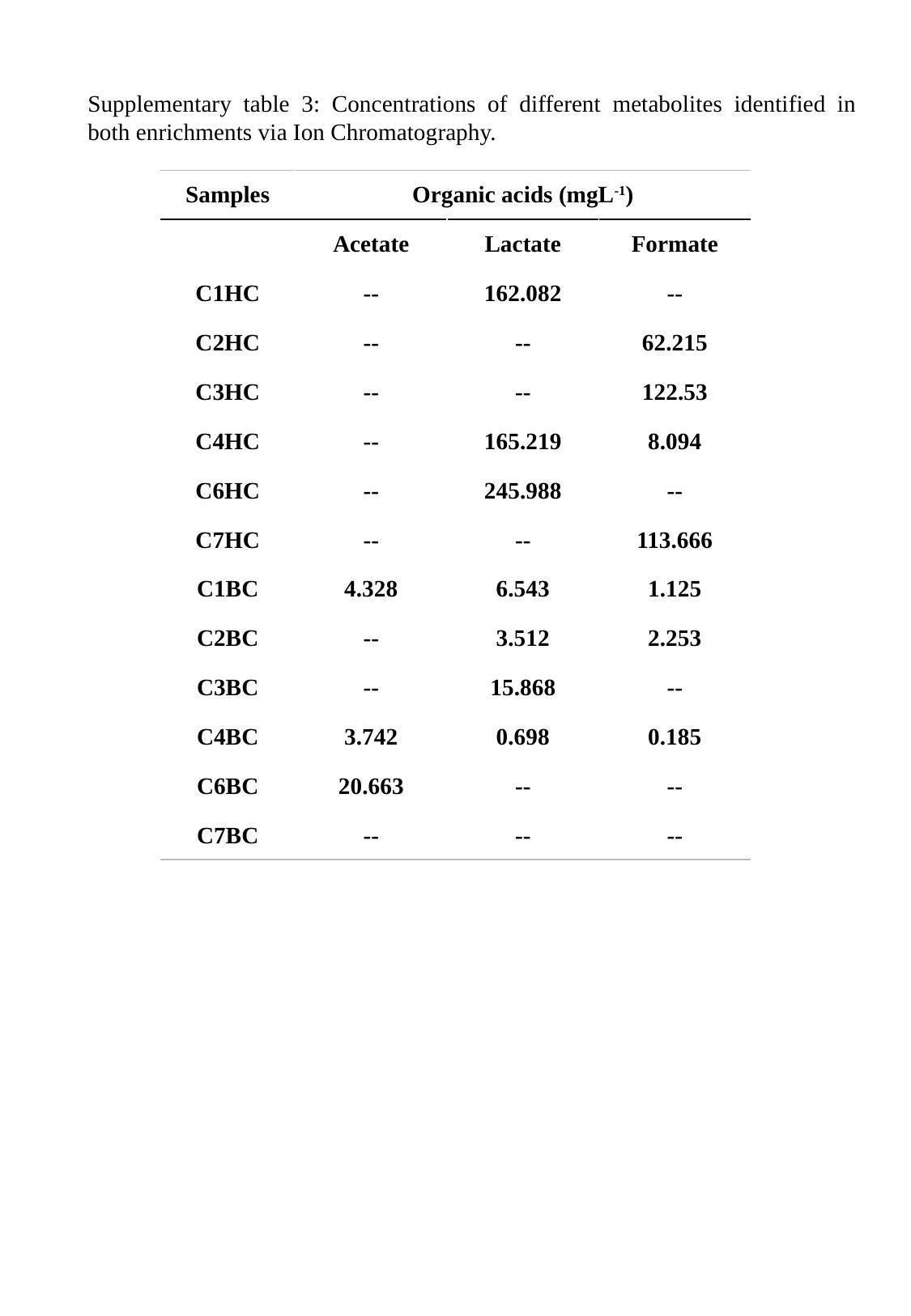

Supplementary table 3: Concentrations of different metabolites identified in both enrichments via Ion Chromatography.
| Samples | Organic acids (mgL-1) | | |
| --- | --- | --- | --- |
| | Acetate | Lactate | Formate |
| C1HC | -- | 162.082 | -- |
| C2HC | -- | -- | 62.215 |
| C3HC | -- | -- | 122.53 |
| C4HC | -- | 165.219 | 8.094 |
| C6HC | -- | 245.988 | -- |
| C7HC | -- | -- | 113.666 |
| C1BC | 4.328 | 6.543 | 1.125 |
| C2BC | -- | 3.512 | 2.253 |
| C3BC | -- | 15.868 | -- |
| C4BC | 3.742 | 0.698 | 0.185 |
| C6BC | 20.663 | -- | -- |
| C7BC | -- | -- | -- |
