# Supplementary material for: Depth wide distribution and metabolic potential of chemolithoautotrophic microorganisms reactivated from deep continental granitic crust underneath the Deccan Traps at Koyna, India
Source: Front Microbiol. 2022 Nov 24;13:1018940. doi: 10.3389/fmicb.2022.1018940 (PMC9731672; doi:10.3389/fmicb.2022.1018940)
Supplement: Supplementary Figure 1 — OTU overlap within HC and BC enrichments to determine the unique and shared OTUs. [file Data_Sheet_2.ZIP › Supp table 4(A,B).pptx]

## Slide 1
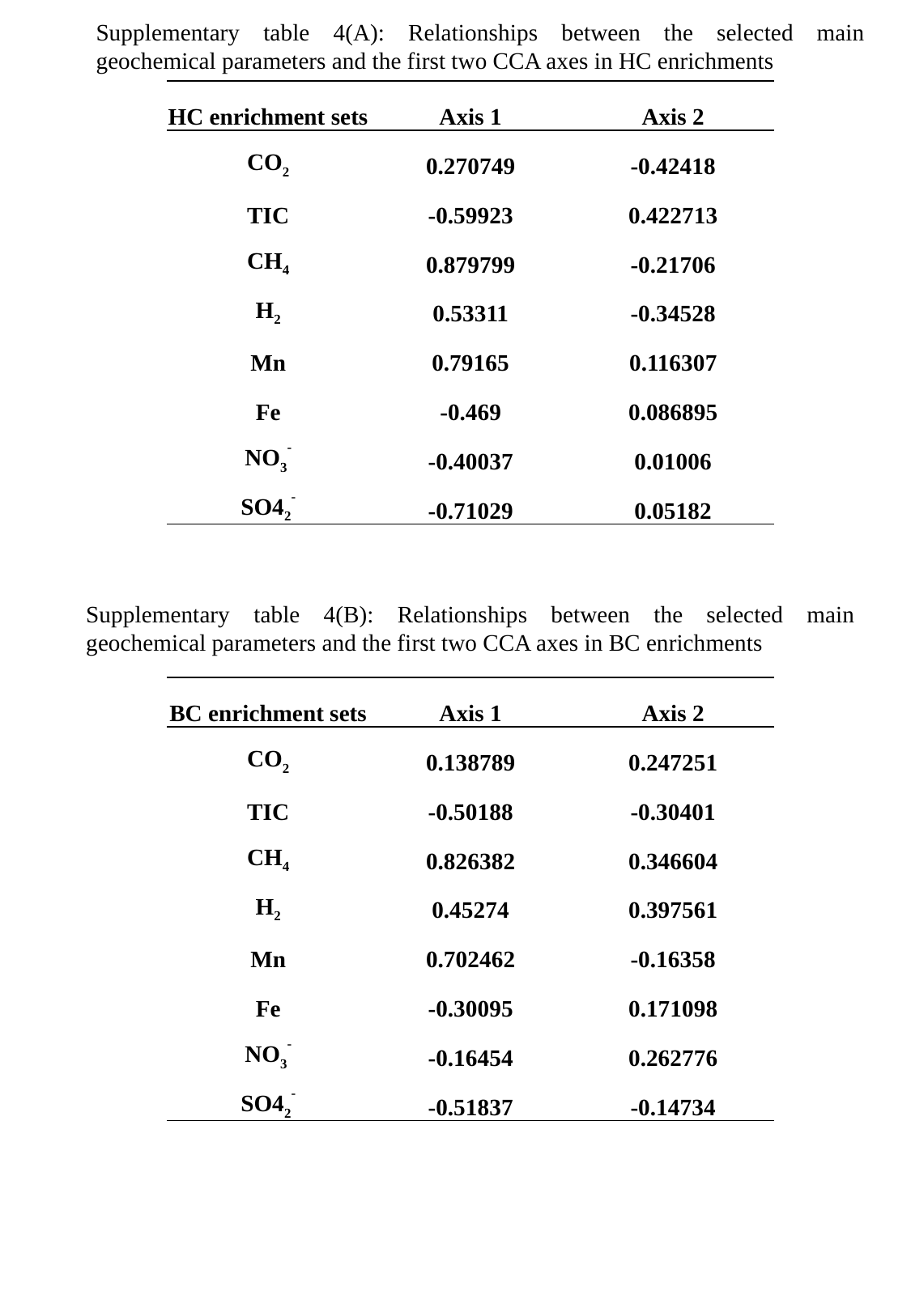

Supplementary table 4(A): Relationships between the selected main geochemical parameters and the first two CCA axes in HC enrichments
| HC enrichment sets | Axis 1 | Axis 2 |
| --- | --- | --- |
| CO2 | 0.270749 | -0.42418 |
| TIC | -0.59923 | 0.422713 |
| CH4 | 0.879799 | -0.21706 |
| H2 | 0.53311 | -0.34528 |
| Mn | 0.79165 | 0.116307 |
| Fe | -0.469 | 0.086895 |
| NO3- | -0.40037 | 0.01006 |
| SO42- | -0.71029 | 0.05182 |
Supplementary table 4(B): Relationships between the selected main geochemical parameters and the first two CCA axes in BC enrichments
| BC enrichment sets | Axis 1 | Axis 2 |
| --- | --- | --- |
| CO2 | 0.138789 | 0.247251 |
| TIC | -0.50188 | -0.30401 |
| CH4 | 0.826382 | 0.346604 |
| H2 | 0.45274 | 0.397561 |
| Mn | 0.702462 | -0.16358 |
| Fe | -0.30095 | 0.171098 |
| NO3- | -0.16454 | 0.262776 |
| SO42- | -0.51837 | -0.14734 |
