# Supplementary figures and images for: Depth wide distribution and metabolic potential of chemolithoautotrophic microorganisms reactivated from deep continental granitic crust underneath the Deccan Traps at Koyna, India
Source: Front Microbiol. 2022 Nov 24;13:1018940. doi: 10.3389/fmicb.2022.1018940 (PMC9731672; doi:10.3389/fmicb.2022.1018940)

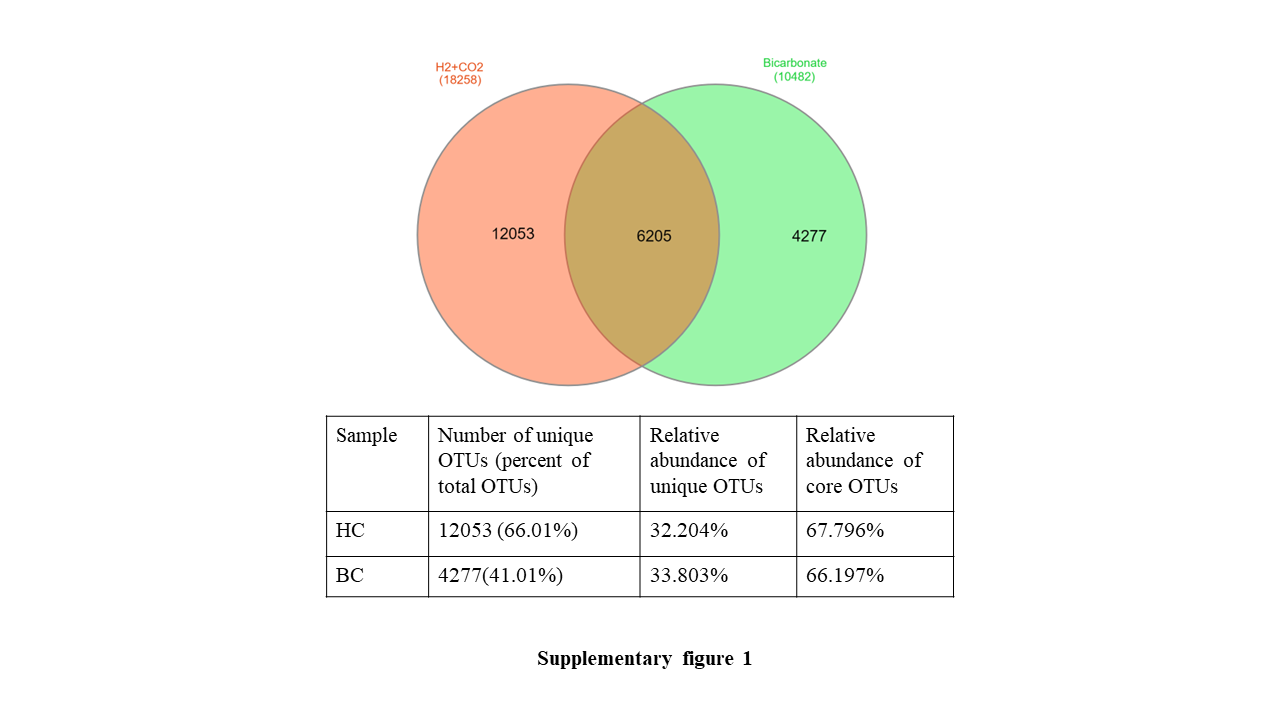

Supplement: Supplementary Figure 1 — OTU overlap within HC and BC enrichments to determine the unique and shared OTUs. [file Data_Sheet_2.ZIP › Supp fig 1.tif]

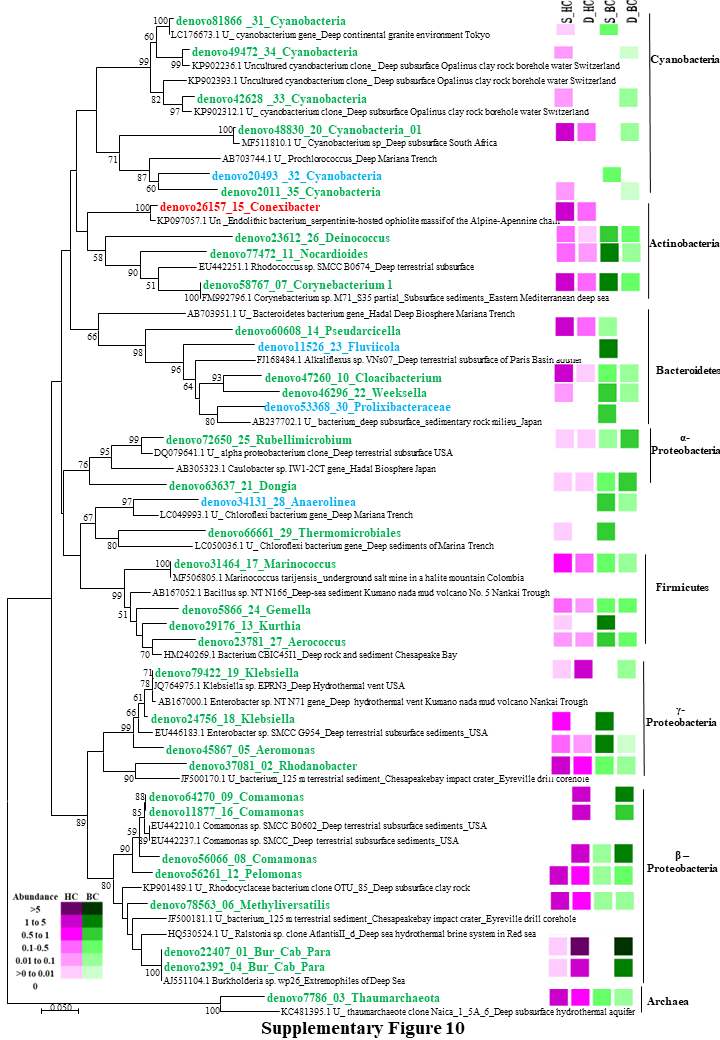

Supplement: Supplementary Figure 1 — OTU overlap within HC and BC enrichments to determine the unique and shared OTUs. [file Data_Sheet_2.ZIP › Supp fig 10.tif]

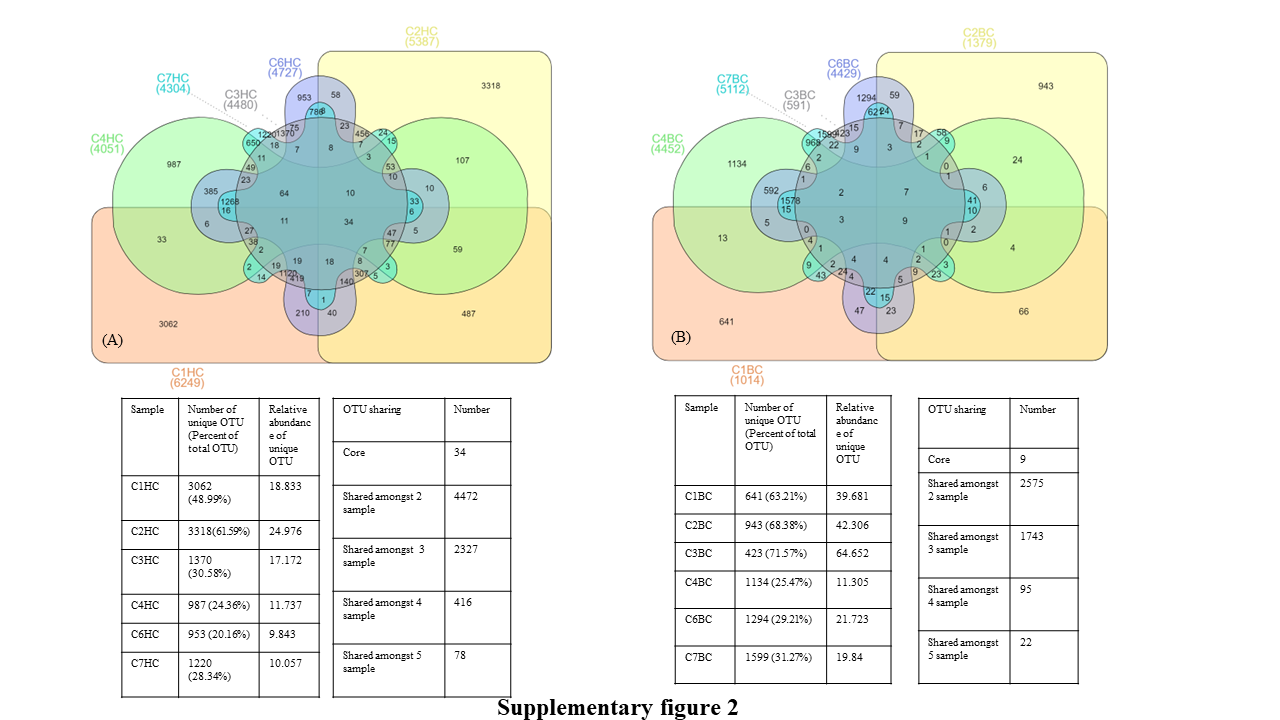

Supplement: Supplementary Figure 1 — OTU overlap within HC and BC enrichments to determine the unique and shared OTUs. [file Data_Sheet_2.ZIP › Supp fig 2(A, B).tif]

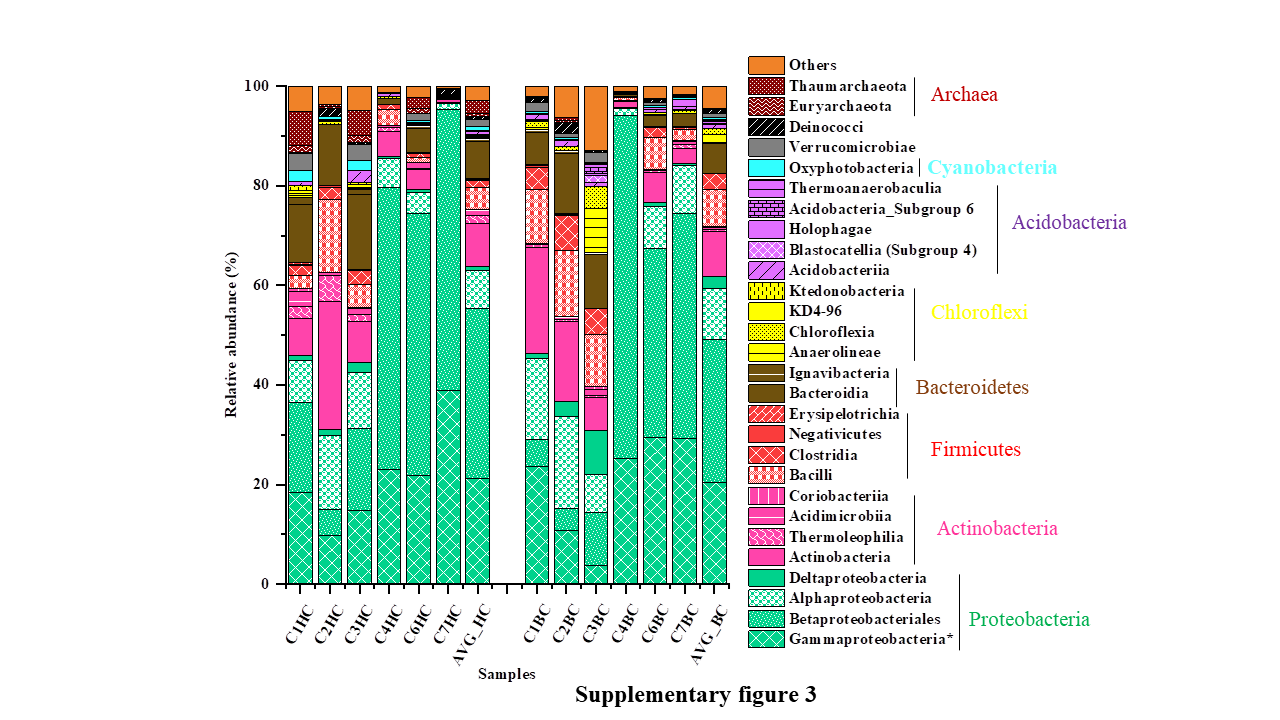

Supplement: Supplementary Figure 1 — OTU overlap within HC and BC enrichments to determine the unique and shared OTUs. [file Data_Sheet_2.ZIP › Supp fig 3.tif]

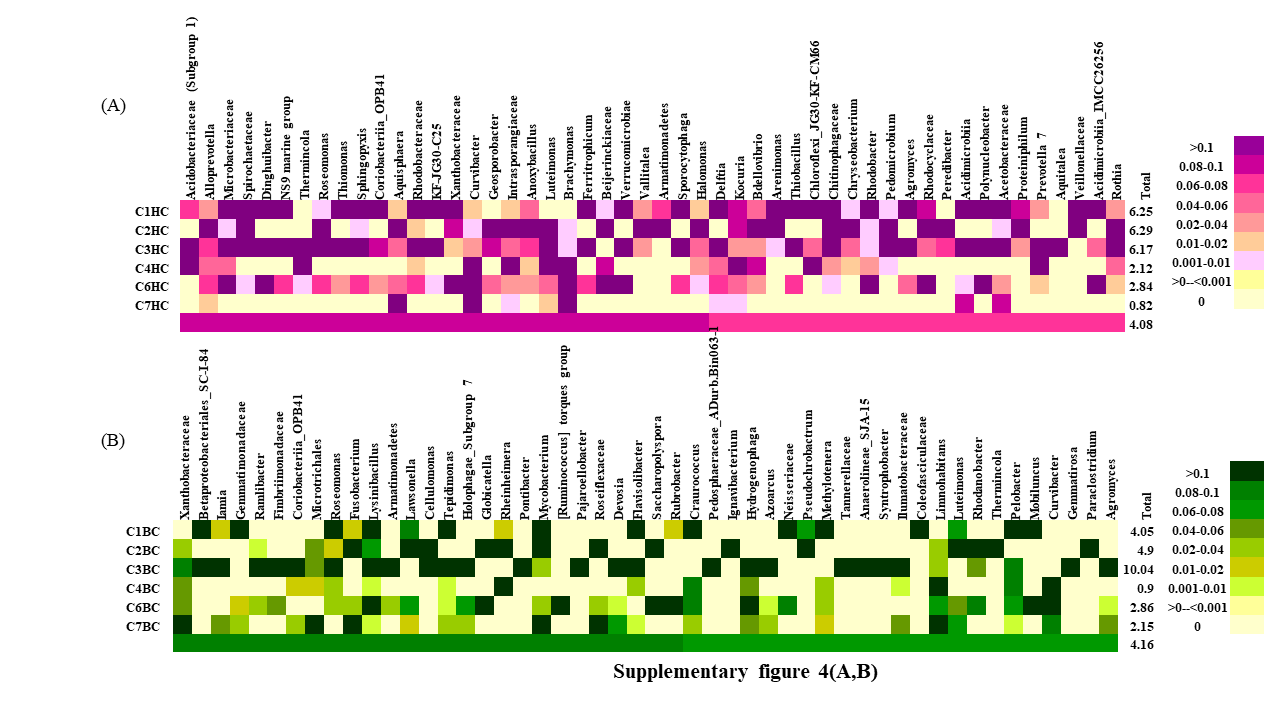

Supplement: Supplementary Figure 1 — OTU overlap within HC and BC enrichments to determine the unique and shared OTUs. [file Data_Sheet_2.ZIP › Supp fig 4(A,B).tif]

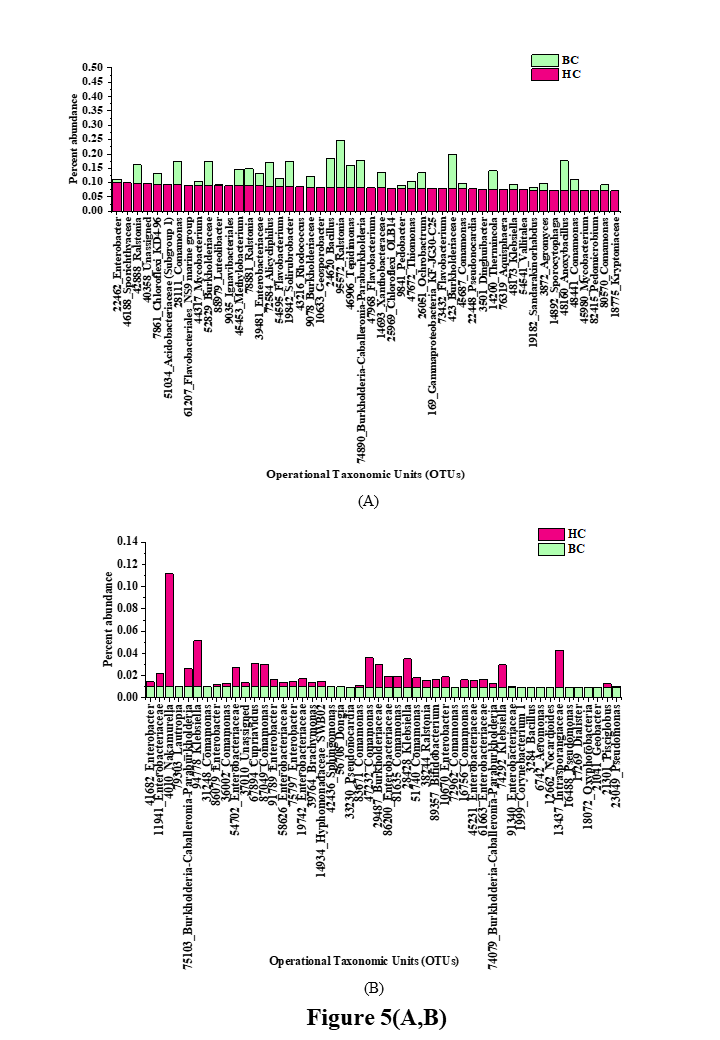

Supplement: Supplementary Figure 1 — OTU overlap within HC and BC enrichments to determine the unique and shared OTUs. [file Data_Sheet_2.ZIP › Supp fig 5(A, B).tif]

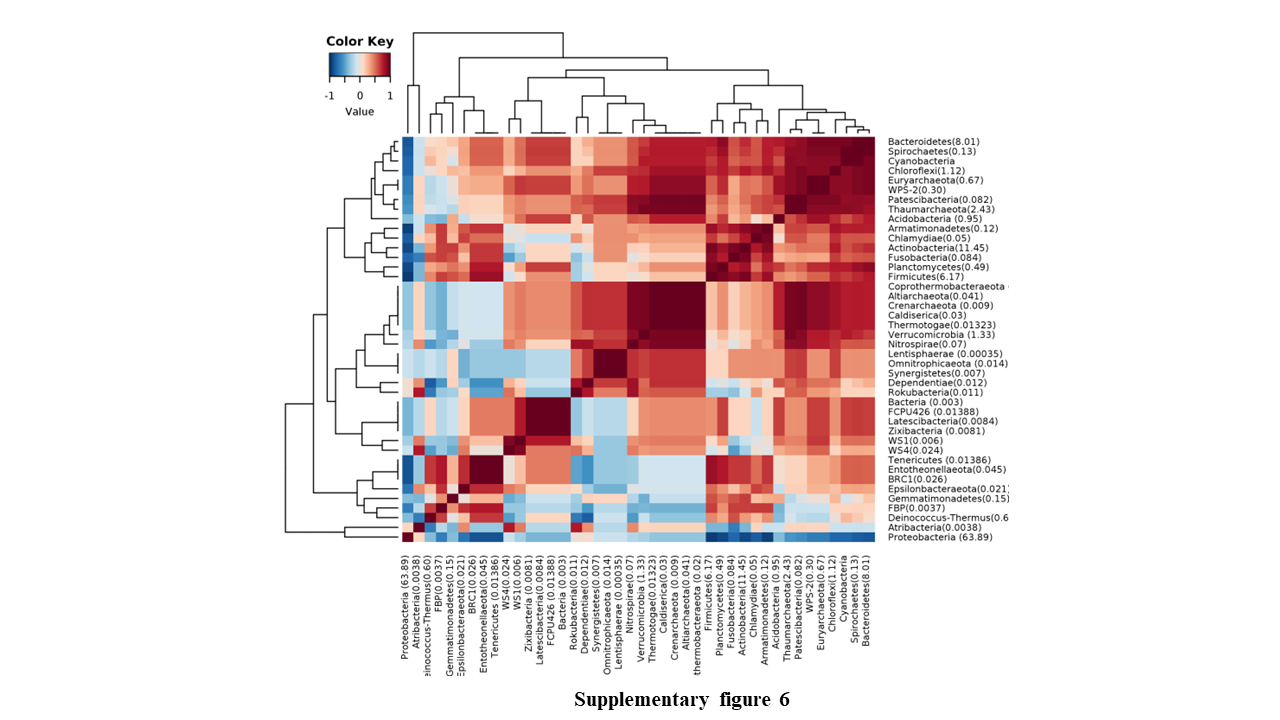

Supplement: Supplementary Figure 1 — OTU overlap within HC and BC enrichments to determine the unique and shared OTUs. [file Data_Sheet_2.ZIP › Supp fig 6.tif]

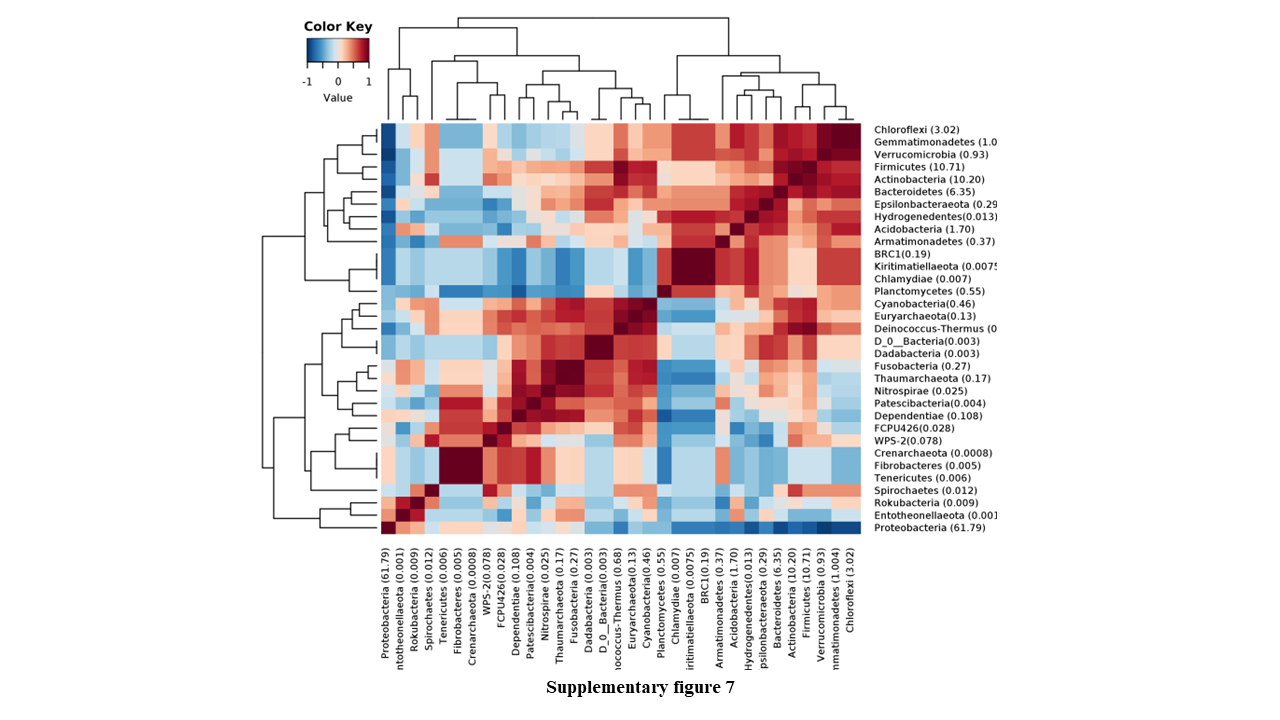

Supplement: Supplementary Figure 1 — OTU overlap within HC and BC enrichments to determine the unique and shared OTUs. [file Data_Sheet_2.ZIP › Supp fig 7.tif]

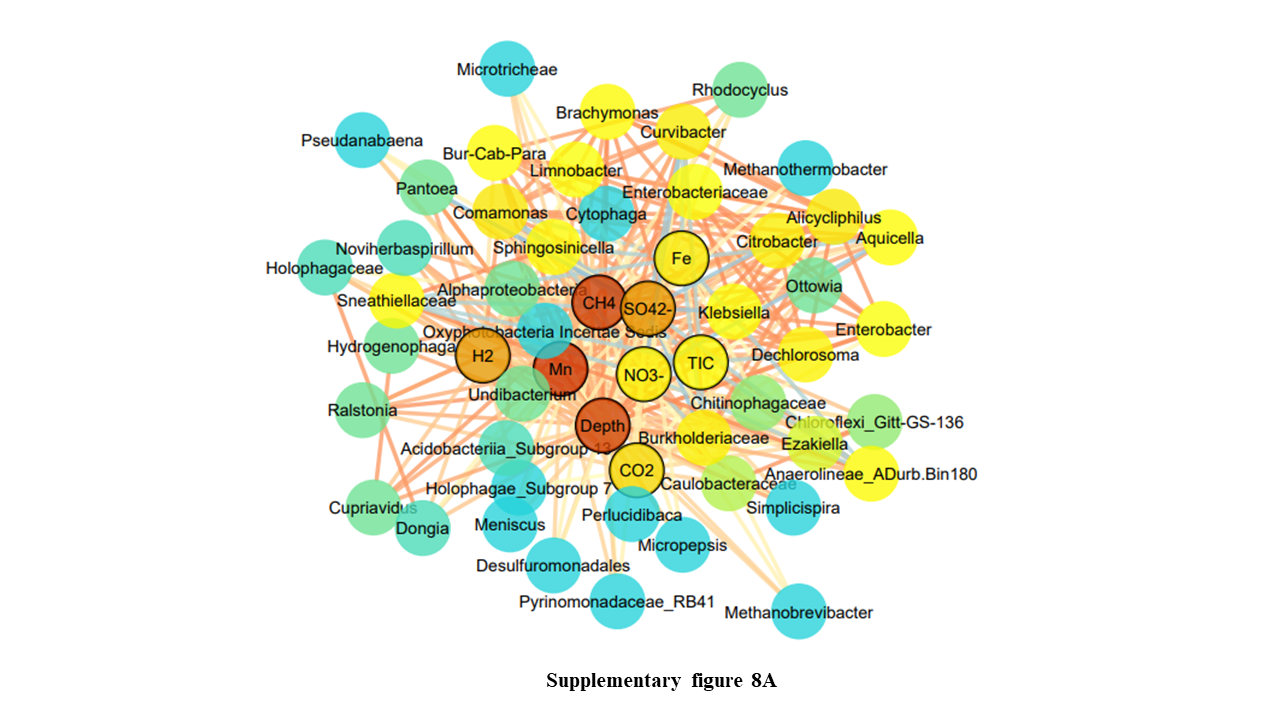

Supplement: Supplementary Figure 1 — OTU overlap within HC and BC enrichments to determine the unique and shared OTUs. [file Data_Sheet_2.ZIP › Supp fig 8A.TIF]

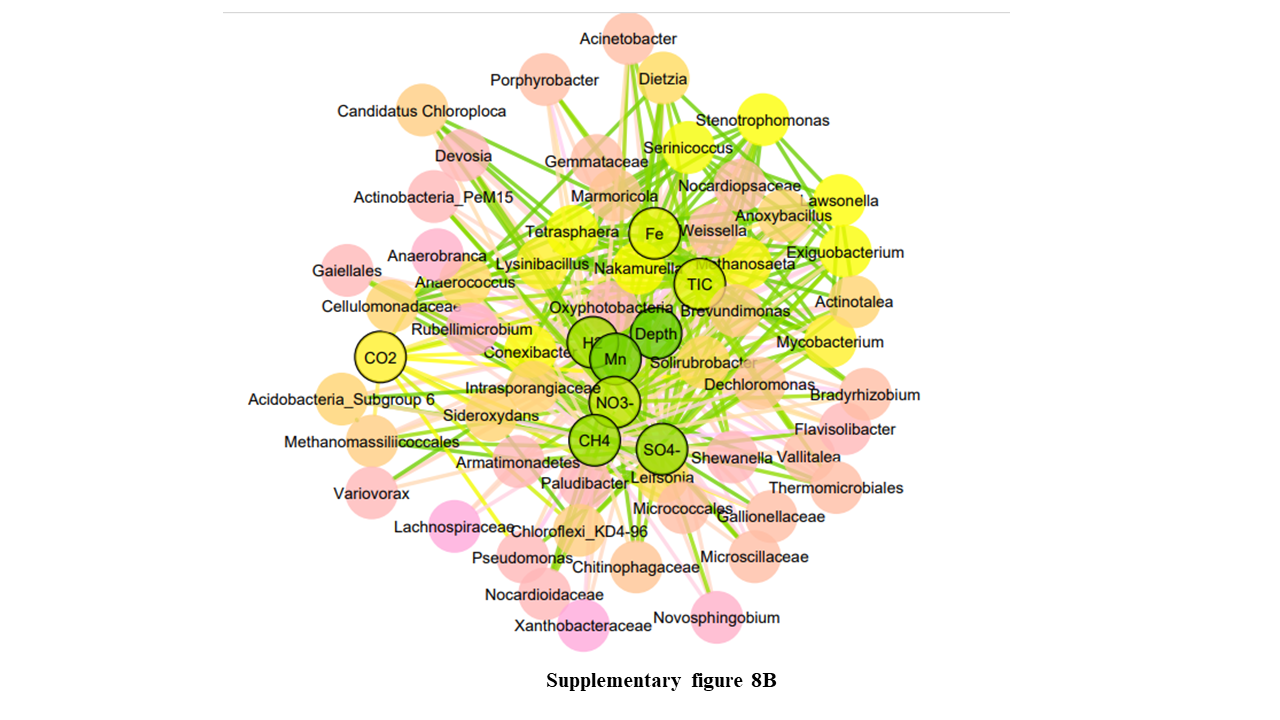

Supplement: Supplementary Figure 1 — OTU overlap within HC and BC enrichments to determine the unique and shared OTUs. [file Data_Sheet_2.ZIP › Supp fig 8B.TIF]

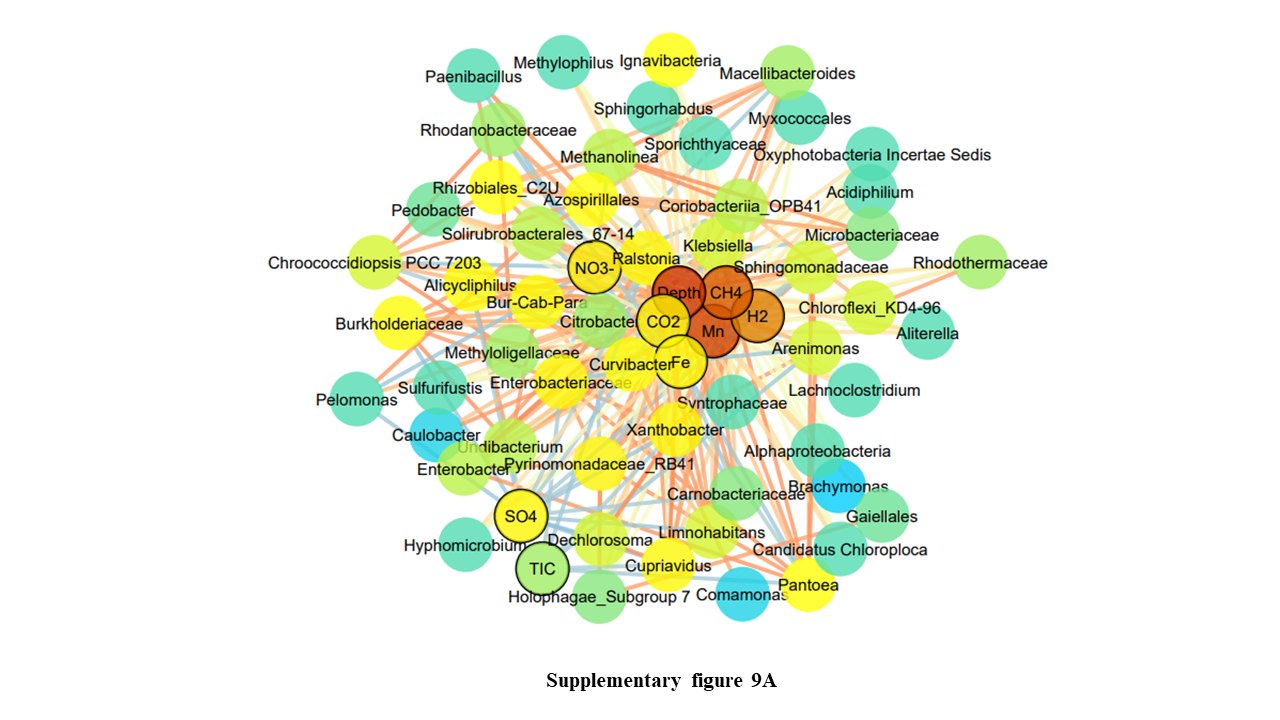

Supplement: Supplementary Figure 1 — OTU overlap within HC and BC enrichments to determine the unique and shared OTUs. [file Data_Sheet_2.ZIP › Supp fig 9A.TIF]

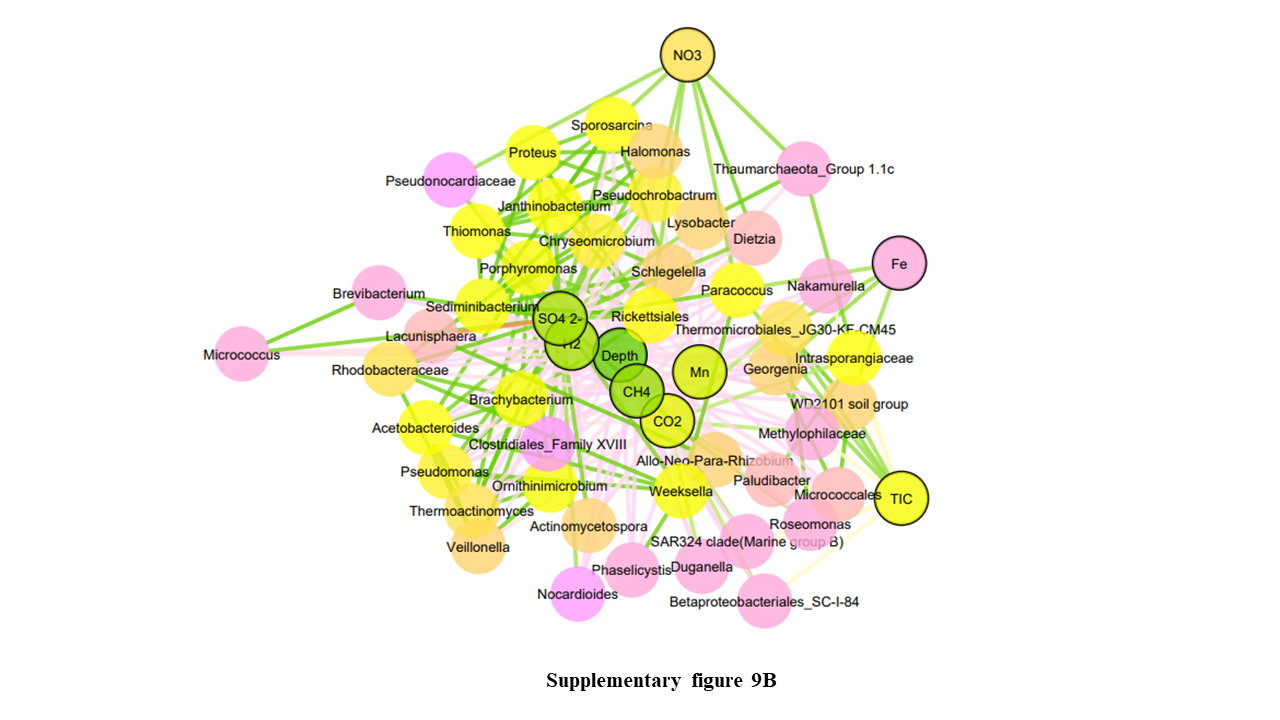

Supplement: Supplementary Figure 1 — OTU overlap within HC and BC enrichments to determine the unique and shared OTUs. [file Data_Sheet_2.ZIP › Supp fig 9B.TIF]
